# VASIR: An Open-Source Research Platform for Advanced Iris Recognition Technologies

**DOI:** 10.6028/jres.118.011

**Published:** 2013-04-23

**Authors:** Yooyoung Lee, Ross J Micheals, James J Filliben, P Jonathon Phillips

**Affiliations:** National Institute of Standards and Technology, Gaithersburg, MD 20899

**Keywords:** benchmark, biometrics, feature extraction, image quality, iris recognition, open-source, research platform, segmentation, VASIR

## Abstract

The performance of iris recognition systems is frequently affected by input image quality, which in turn is vulnerable to less-than-optimal conditions due to illuminations, environments, and subject characteristics (e.g., distance, movement, face/body visibility, blinking, etc.). VASIR (Video-based Automatic System for Iris Recognition) is a state-of-the-art NIST-developed iris recognition software platform designed to systematically address these vulnerabilities. We developed VASIR as a research tool that will not only provide a reference (to assess the relative performance of alternative algorithms) for the biometrics community, but will also advance (via this new emerging iris recognition paradigm) NIST’s measurement mission. VASIR is designed to accommodate both ideal (e.g., classical still images) and less-than-ideal images (e.g., face-visible videos). VASIR has three primary modules: 1) Image Acquisition 2) Video Processing, and 3) Iris Recognition. Each module consists of several sub-components that have been optimized by use of rigorous orthogonal experiment design and analysis techniques. We evaluated VASIR performance using the MBGC (Multiple Biometric Grand Challenge) NIR (Near-Infrared) face-visible video dataset and the ICE (Iris Challenge Evaluation) 2005 still-based dataset. The results showed that even though VASIR was primarily developed and optimized for the less-constrained video case, it still achieved high verification rates for the traditional still-image case. For this reason, VASIR may be used as an effective baseline for the biometrics community to evaluate their algorithm performance, and thus serves as a valuable research platform.

## 1. Introduction

Biometrics is the method for identifying an individual based on human physiological or behavioral characteristics. Biometric-based technologies are increasingly being incorporated into security applications—for example, banking, border control, access control, law-enforcement, and forensics [1]. One biometric of particular interest is to use the human iris due to its being highly distinctive and resulting high accuracy of identification. Iris recognition is the method for identifying a person based on the highly distinctive patterns of the human iris.

Despite the generally high accuracy of iris recognition systems, some users found such systems demanding in terms of head/eye positioning, camera positioning, and time taken in the enrollment process. This drawback was especially exacerbating for participants who were physically disabled [2]. In addition to this usability issue, iris recognition systems may be limited by input and environmental factors which affect image quality. For example, an iris recognition system might perform well only under ideal imaging conditions, such as when the iris image was captured with a camera specifically designed for the system. Furthermore, most iris-based biometric systems, especially commercial products, carry out verification/identification studies based primarily on *traditional* still iris images captured in highly controlled environments with fully cooperative subjects. “Traditional still iris images” are iris images captured by devices designed specifically for iris recognition, for example, the LG2200^1^ and LG4000^1^. For the broader authentication problem, subjects may not always be cooperative (for example, law enforcement and military applications) and hence this traditional still-based iris recognition may be infeasible or certainly less-than optimal.

To address scenarios where still-based methods are inappropriate, a *video-based* approach is a promising, although challenging, alternative. Video-based (non-ideal) iris analysis consists of capturing iris features while a person is in motion, usually at a distance, and under less-constrained environments. Given this, video-based iris recognition has the potential to be used in a much broader scope of applications and environments.

Further, various matching scenarios using multiple imaging sources—different media types (e.g., ideal still image to non-ideal video)—are even more promising. This approach however needs to deal with a number of issues such as acquiring an iris image from a distance, poor illumination, off-angle viewing, varying iris resolution, subject movement, blinking, and isolating the iris region from a face/body visible video. The VASIR (Video-based Automatic System for Iris Recognition)^2^ platform includes means for compensating for such challenges. Other challenging aspects that this research tool addresses include video sequences of a subject walking through a portal, performing iris recognition of a person in a face-visible sequence, extracting ocular sub-images, extracting iris sub-images, coping with high and low resolution, adjusting for poor imaging condition, and matching such images to multi-media archival imagery.

The primary goals of this paper are to introduce VASIR and to illustrate its use and scope to serve as a research baseline for promoting iris-based biometric technology. The VASIR system was developed to enhance verification performance by addressing the above-described challenges via a rigorous and structured design and analysis methodology. The motivation for VASIR’s development is to provide an open-source computational research tool that addresses:

1. image acquisition: constrained and less-constrained,
2. image condition: ideal and non-ideal, and
3. image type: still and video imagery.

The VASIR system has three modules: 1) Image Acquisition, 2) Video Processing, and 3) Iris Recognition. The Image Acquisition module was designed to have the capacity to load both still-images and video-sequences. The goal in designing the Video Processing module was to develop an automatic system to effectively deal with videos that contain extraneous features (e.g., face, hair, ear, shoulder). Sub-components are eye region detection, iris image extraction, image quality measurement, and best quality image selection. The goal in designing the Iris Recognition module was to develop (and robustly optimize) an automatic algorithm with better performance than current open-source systems. Sub-components are segmentation, normalization, feature extraction/encoding with a new masking scheme, and similarity distance metrics with an enhanced bit-shifting method.

Some of VASIR’s algorithmic parameters were optimized in a statistically rigorous fashion by using orthogonal fractional factorial experiment designs. Details of this extensive optimization and sensitivity analysis study are given in [3,4].

To provide the necessary performance benchmark baseline, we evaluate VASIR’s performance with multiple datasets that were taken by multiple media sources under a variety of imaging conditions. In terms of utility, the VASIR platform will be valuable research tool for NIST, academia, industry, and government in the following ways:

1. Challenge Problems: NIST designs “grand challenges” to facilitate and support algorithm and technology development by industry and academia alike. Part of the infrastructure to initiate such grand challenges includes the existence of working examples, scoring packages, and baseline algorithms such as VASIR.
2. Research Baselines: Reference implementations like VASIR are based on readily available algorithms and well-established techniques from the scientific literature so as to provide researchers the ability to rapidly assess the viability of alternative approaches and component technologies.
3. Open Algorithms and Standard Components: Commercial products are complex highly-engineered “black-box” systems. To design effective performance metrics and methods for such systems, an open research algorithm is extremely useful in designing technology evaluations. VASIR’s open-source algorithms facilitate the development of testing protocols around standardized components. Further, such open algorithms are educational not only to NIST, but also for academia, industry, and government researchers.

In summary, the iris-based research and academic community needs the ability to access advanced iris recognition software in which component algorithms can be examined, source code can be re-used, and critical performance bottlenecks can be identified. VASIR’s open framework makes it an ideal research tool/resource for the biometric community to examine the effect of algorithmic component changes, to extract and re-use the source code, to carry out comparative studies on common reference datasets and to otherwise advance the state-of-the-art of iris recognition technology.

## 2. The VASIR System Overview

Many commercial systems for iris recognition are computational black boxes that run proprietary internal algorithms. In this light, IrisBEE (Iris Biometric Evaluation Environment) [5] was implemented in 2005 in C language from Masek’s Matlab code [6] and fulfills an important role in the iris recognition community since the system is both non-commercial and open-source. Despite its significance and impact—since IrisBEE was developed to address the traditional still-based iris recognition problem—there is a current need to overcome a number of challenges when images are taken under the flexible range of conditions encountered in the real world (e.g., face-visible video taken at a distance while a person walks through a portal).

To advance iris-based biometrics technology, we developed VASIR with Near-Infrared (NIR) face-visible *video*-based iris recognition as part of its focus and scope. VASIR is a fully-automated research platform for video-based iris recognition, capable of handling videos that were 1) captured under less-constrained environmental conditions and 2) contained other features (e.g., face, head, hair, neck, shoulder) apart from the eye/iris region, such as a person walking through a portal at a distance. The VASIR system was designed, developed, and optimized to be robust—to address the challenge of recognizing a person using the human iris in less-than-optimal environments, coping with both high and low image/video quality. Although VASIR was developed with less-constrained video-based iris recognition in mind, VASIR also robustly accommodates traditional constrained still-based iris recognition. VASIR supports multiple matching scenarios:

1. less-constrained (non-ideal) video to less constrained (non-ideal) video (VV),
2. less-constrained (non-ideal) video to constrained (ideal) still image (VS), and
3. constrained (ideal) still to constrained (ideal) still image (SS) iris recognition.

In VV matching, the extracted iris region of video frames taken at a distance is matched to other frames from the same video, or to frames from a different video sequence of the same person. The VS matching scenario means that the video frame (e.g., taken at a distance using an Iris On Move (IOM)^1^ system) is compared to classical still-images (e.g., via a LG2200^1^ system)—matching samples captured by a different device. SS matching is the traditional iris recognition scenario in which a classical still image is matched against other classical still images of the same person—all of which were captured by the same device.

VASIR has the capacity to load both still images and the video sequences, to automatically detect and extract the eye region, and to subsequently assess and select the best quality iris image from NIR face-visible video captured at a certain distance. After this process, VASIR carries out a comprehensive image comparison analysis that in turn yields a state-of-the-art subject verification.

**Fig. 1:**
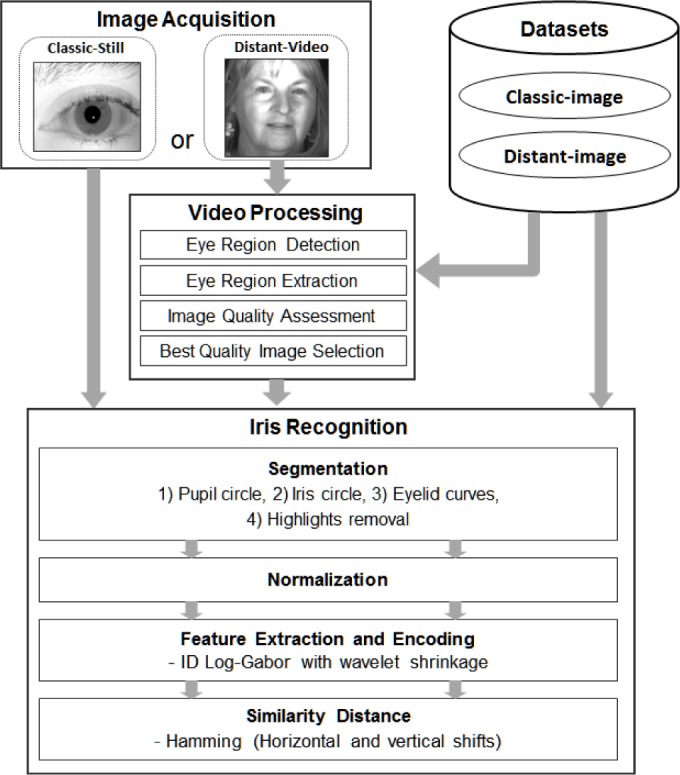
VASIR system for both classic-still and distant-video.

As shown in Fig. 1, the VASIR system has three primary modules: 1) Image Acquisition, 2) Video Processing, and 3) Iris Recognition.

Each module consists of several sub-components that have all been designed, developed, and optimized to achieve high verification performance. In the *Image Acquisition* module, VASIR loads the image or video sequence. As input for evaluation, we use the existing publicly available datasets ICE2005 [5] and MBGC [9] that will be described in Sec. 3. In the *Video Processing* module, VASIR automatically detects the eye region and separate it from face, hair, ear, and shoulder that may be visible in video frames, and subsequently extracts the left/right iris sub-images separately. VASIR then calculates an image quality score of the extracted iris sub-images. Based on the quality score, the best quality iris images—one for left and one for right—are chosen out of all available frames. The *Iris Recognition* module is then fed either the resulting iris images from the Video Processing module or the loaded still image. For both video and still iris images, VASIR segments the iris region based on its own segmentation approach. The segmented iris regions are then extracted and normalized into a polar representation using bilinear interpolation. Next, VASIR extracts the features from the normalized iris images and encodes the extracted features as binary strings along with a VASIR-developed noise-masking scheme. At the end, VASIR matches the encoded biometric iris template to other existing iris templates. Note that the procedures of all three modules are completely automatic.

## 3. The VASIR Image Acquisition Module

The performance of iris-based biometric systems is directly related to iris image quality. The image quality is normally determined at the image collection stage and is influenced by camera type, environmental conditions, subject behavior/characteristics, among others. In general, higher quality images lead to higher performance results for a system. Since the image acquisition process is important, there is a need for consideration of what constitutes optimal quality image collection and how that relates to the usability and effectiveness of the system.

Most traditional iris acquisition systems provide a relatively good quality image for the purpose of iris recognition; on the other hand, such systems typically require heavy cooperation on the part of the subject to make necessary adjustments for proper distance to the camera, for proper eye position, and for proper head position. Such image acquisition requirements are slow and difficult to interact with. Further, such imaging is not practical—especially in a security scenario where the subject may be distant and may not always be cooperative.

The image acquisition system process has been steadily improved by researchers [10], attempting to make the process more effective and user-friendly. Nevertheless, there are still a number of ongoing challenges—which our study addresses. Hence, VASIR deals with both the traditional still iris images and the more challenging face-visible video sequences.

Two popular acquisition systems that use different acquisition approaches are discussed in the sections below.

### 3.1 Image Acquisition Systems

#### A. LG 2200 system (“Classic-Still”)

**Fig. 2:**
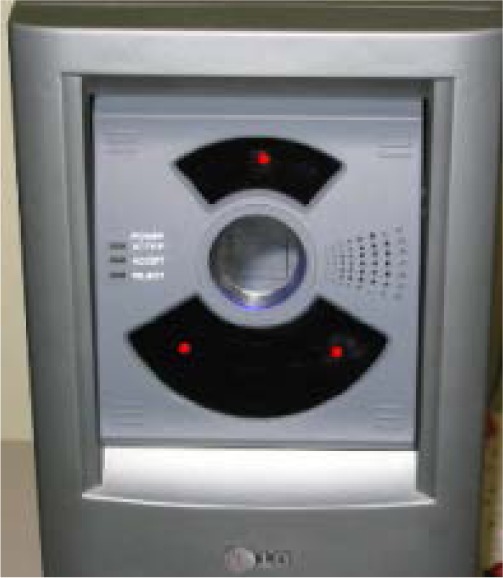
A LG2200 System with three LED illuminators—taken from the paper written by Bowyer and Flynn [11] with permission.

The LG 2200 system [11] captures classic-still iris images and interacts with the subject by providing vocal instruction to guide the subject: the system will ask the user to adjust his/her distance to the sensor, eye position, and head position properly during the image acquisition process. The iris is illuminated by three near-infrared (NIR) LEDs, which appear as red dots in Fig. 2. Specular highlights from these illuminators are visible in the pupil region within an image.

The LG2200 captures and produces three iris images and selects one of them with its built-in image quality control software. The iris images are 640×480 pixels in resolution and the iris diameter for optimal images typically exceeds 200 pixels. All images are stored in 8 bits of gray-scale.

#### B. IOM: Iris On Move (“Distant-Video”)

**Fig. 3:**
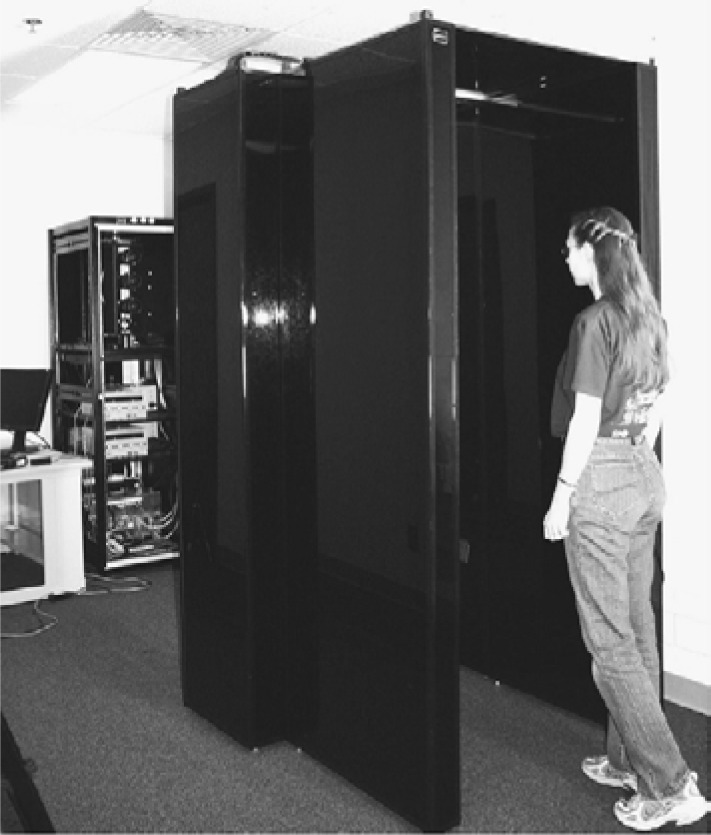
Portal acquisition process at a distance by an Iris On the Move (IOM) system—taken from the paper written by Bowyer *et al.* [10] with permission.

According to a study conducted by Atos Origin for the U.K. Passport Service [2], iris recognition was the most preferred biometric method for the participant—compared to fingerprint and face recognition. Nevertheless, the participants found it difficult to interact with the system, especially positioning and the time taken in the enrollment process, particularly for disabled participants. To address these concerns, Matey *et al.* [12] developed the Iris On Move (IOM) system to provide a decreased acquisition time and to make the capturing process easier. The system has the capability of taking a NIR face-visible video of subjects while they are walking through a portal at normal walking speed at a distance (approximately 10 feet [3m] away) as shown in Fig. 3. The IOM output will be videos consisting primarily of the face, hair, ear, and neck.

The portal itself contains NIR lights to illuminate the subject. Subjects look forward while they walk and subjects are allowed to wear normal eye-glasses and contact lenses, but not sunglasses [10]. Videos have a high resolution (2048×2048 pixels) for each frame and the iris diameter varies (average size ~100 pixels).

### 3.2 Datasets for Benchmark Evaluation

For the purpose of the benchmark evaluation, we examine the VASIR system performance using publically available datasets collected by ICE (Iris Challenge Evaluation) and MBGC (Multiple Biometric Grand Challenge).

#### A. Iris Challenge Evaluation (ICE) 2005

The ICE2005 dataset [5] is a traditional NIR iris still image set captured by an LG2200 acquisition system which we call “*classic-still*” iris images. Table 1 illustrates the number of images and subjects in the ICE 2005 dataset.

**Fig. 4:**
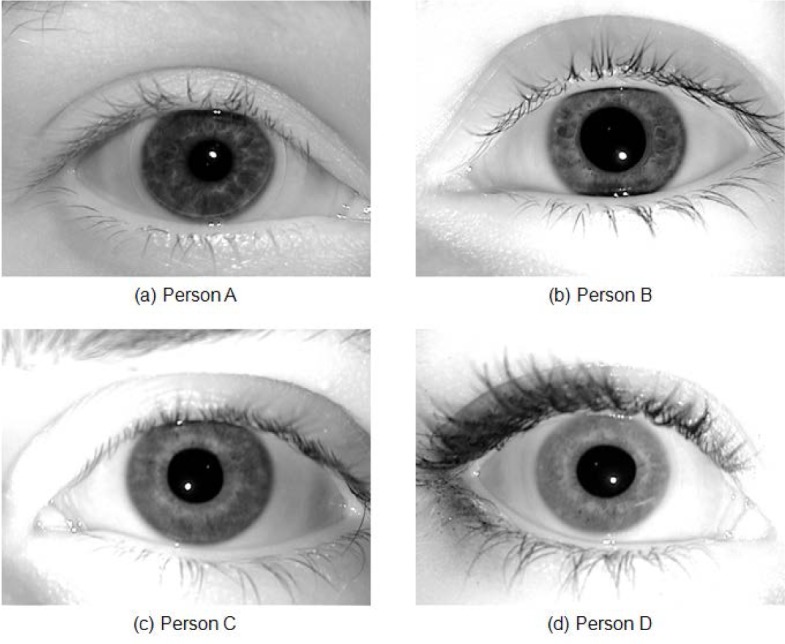
Examples from the ICE2005 dataset (640×480 resolution in grayscale).

The total number of iris images for both left and right eyes is 2,953 on 132 distinct subjects. Some subjects (120) are left eye only, some (124) right eye only, and most (112) have both eyes. Figure 4 illustrates sample images from the ICE2005 dataset.

All images in the ICE2005 dataset have 640×480 pixel resolution. As mentioned above, the LG2200 system normally selects one image out of three images that were taken in a shot, and discards the other two images using a built-in image quality control system. This ICE2005 dataset, however, contains all three images to support a broad range of image quality for iris-based biometrics research. Therefore, one image out of three meets the LG2200 built-in quality checks, while the other two images may or may not meet the quality check requirements. As an aside, note that the original ICE2005 dataset mistakenly included the image “246260.tiff” in the left image set instead of the right image set, and consequently we excluded this image from our study—see details in the paper written by Phillips *et al.* [5].

#### B. MBGC (Multiple Biometrics Grand Challenge)

The Multiple Biometrics Grand Challenge (MBGC) dataset [9] includes iris images under varying illumination conditions, low quality, and off-angle (or occluded) images in both still images and video sequences.

NIR face-visible video (distant-video) datasets from the MBGC are used for evaluations in this study; the distant-video samples were captured with a video camera by the Sarnoff IOM system in 2048×2048 resolution; with face, hair, neck, and shoulder visible in the screen—note that the size of the eye region sub-image varies depending on the subject’s distance and movement toward the camera.

The MBGC dataset can be divided into two versions; version 1 is almost a proper subset of version 2. Due to its smaller size, we used version 1 for evaluating the segmentation component under the Iris Recognition module. For all remaining VASIR component evaluations, we used the larger dataset version 2. Table 2 summarizes both versions of the MBGC distant-video dataset.

For version 1, there are a total of 149 video sequences for distant-video collected from 114 subjects by IOM. Of the 149 sequences, there is no eye visible in just one of the video sequences, and in 18 other video sequences only one eye is visible.

**Fig. 5:**
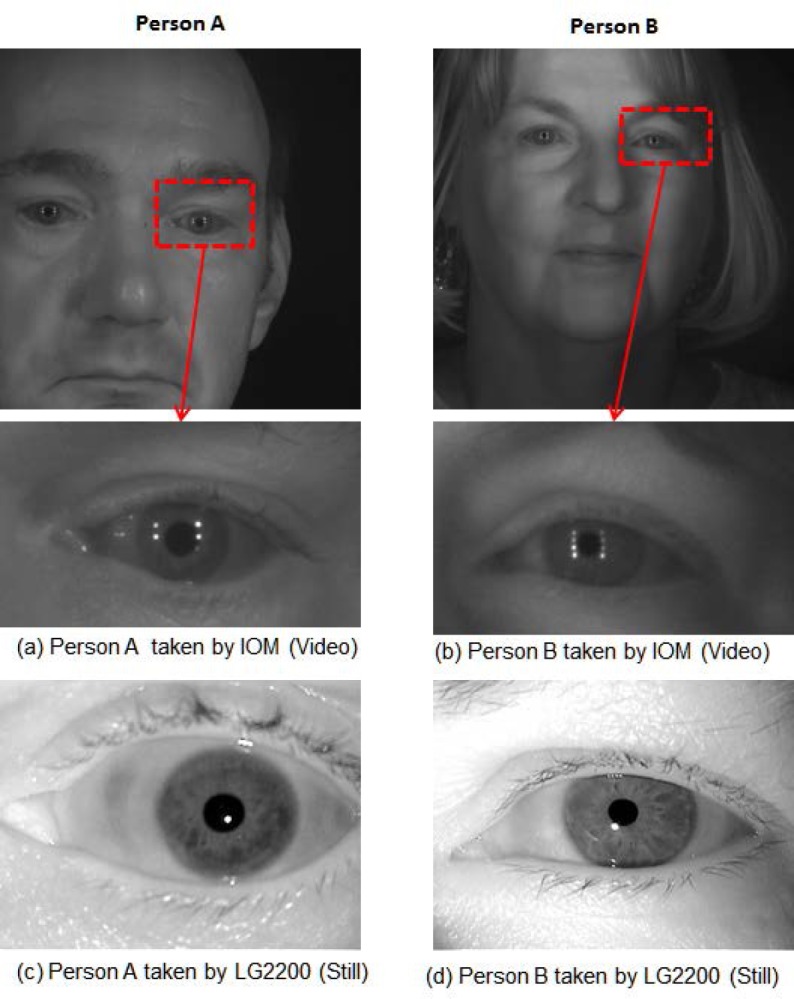
Quality differences between (a)-(b) sub-image extracted from *distant-video* taken by IOM and (c)-(d) *classic-still* taken by LG2200; (a) and (c) are from person A, while (b) and (d) are from person B.

For version 2, there are a total of 628 video sequences collected from 143 subjects. Out of the total of 628 videos, there are 30 videos in which either no eye is visible or only one eye is visible. Some subjects exist in only one of the video sequences; others exist in multiple video sequences—up to 10. Distant-video data is normally considered to be a poor-quality image source since the video was taken with moving subjects at a distance, having motion blur, poor contrast, off-angle, poor illumination, and other deficiencies. In contrast, classic-still images have considerably better quality since the acquisition device contains the image quality control software itself and the system limits the users’ behavior and distance during the acquisition procedure. Figure 5 shows an example of the clear quality difference between distant-video (top) and classic-still (bottom) iris images. Figure 5 (a) and (b) show the distant-video sample and the sub-image (eye region) extracted from that frame and Fig. 5 (c) and (d) show the classic-still iris image sample. Figure (a) and (c) are for person A, while (b) and (d) are from person B—note quality differences between the distant-video (top) and classic-still images (bottom).

## 4. The VASIR Video Processing Module

Although distant-video is frequently considered to be a poor-quality image source, the advantage of using these videos is that they can potentially yield more information since many images are captured in one video (15 per second); thus, there is a far broader choice of images from which to choose for the matching process.

On the other hand, the difficulty that many iris-based systems have when dealing with videos—aside from the poorer image quality—is that such videos frequently contain other unimportant features (e.g., face, head, hair, neck, shoulder) which make localizing the iris region more challenging.

The Video Processing module in VASIR is designed to overcome these stumbling blocks when dealing with video sequences that contain extraneous features beyond the eye region. VASIR is a fully automated system that detects the eye region, extracts the iris images from face-visible video, measures iris image quality, selects the best quality image in a sequence, and delivers the image to the next step in the process. The Video Processing module consists of four sub-components: Eye Region Detection, Eye Region Image Extraction, Image Quality Measurement, and Best Quality Image Selection.

### 4.1 Automated Eye Region Detection

In the distant-video sequences contained within the MBGC dataset, frames contain the subject’s face and less frequently, his/her neck and shoulder. Since our interest in this study is only the eye area, we apply a method to detect the eye region automatically. The term “eye detection” will be used to refer to the process of the human eye area detection in video streams. The pupil, iris, sclera, eyelids, eyelashes, and eyebrows are features considered to be part of the eye area.

Although many methods for object detection have been suggested in the literature [13–15], object detection based on a learning mechanism became our first choice in developing the eye detection algorithm for VASIR. This method distinguishes an object-of-interest automatically by being trained with a set of varying object samples, i.e. by means of a supervised learning mechanism.

One popular learning-based object detection approaches in a learning mechanism is based on the Adaboost framework [16] and was employed by Viola-Jones [14]. This method was later improved by Lienhart-Maydt [15] through the addition of rotated Haar-like features; this is the method we adapted for the VASIR system.

The most compelling reason for using these Haar-like features is that a feature-based system is computationally less expensive than most exhaustive search algorithms. A two-rectangle feature calculates the difference between the sum of the pixel intensities within two rectangular regions. A three-rectangle feature computes the difference between the sum of the center (black) rectangle and the sum within two outside (white) rectangles. The integral image representation—also known as the summed area tables—utilized in the method allows a rapid computation for rectangle features. The resulting features are then filtered by learning classification functions that are based on the discrete Adaboost algorithm. The Adaboost algorithm uses the training data to construct a strong classifier as a linear combination of weak classifiers.

For the detection process, a cascade is used to degenerate a decision tree at each stage. A cascade means that a positive result from the first classifier is then passed on for a second classifier evaluation. A positive result from the second classifier is passed on for a third classifier evaluation, and so on. A negative result at each stage leads to immediate rejection. A classifier is a cascade of sub-classifiers working with Haar-like features—see more details in Viola-Jones [14]. The classifier is re-sizable so that it can find objects-of-interest independent of size, i.e., the classifier is applied repeatedly at different scales to scan for the object at various sizes.

The OpenCV [7,17] community produced a collection of public domain classifiers; the performance of these classifiers has been evaluated by Castrillon *et al.* [18,19]. To detect both the left and right eye regions—also called the eye-pair—in a face-visible video stream, we selected the eye-pair classifier due to its superior fitting properties, its faster speed, and its scale invariance (handling eye-pair regions of varying sizes). The eye-pair classifier is applied to each frame to find the position and size of both eye regions. The classifier in the cascade is 45×11 pixels in size and consists of 19 stages.

To lower the number of false positives, we found it necessary to define a minimum size for the eye-pair area within frames—we set that minimum size to be 200×45 pixels with the multiple scale factor (average eye-pair detection speed 1.3 ms per frame). For analysis purposes, the detected region is automatically saved into three separate files: eye-pair, left-eye-only and right-eye-only. We evaluate the eye detection algorithm of VASIR using the MBGC distant-video dataset as shown in Table 3.

Out of the 598 videos, the eye-pair was correctly detected in 586 videos. As shown in Table 3, the successful detection rate was 98 % and the failed detection rate was 2 %.

### 4.2 Automated Eye Region Image Extraction

**Fig. 6:**
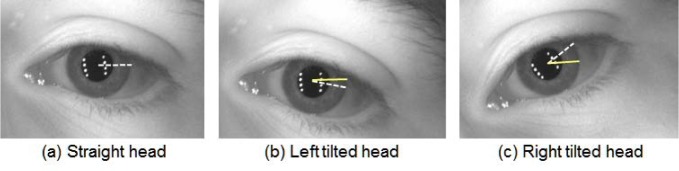
The (a) picture shows the white dotted line with the straight head. The white dotted line locations differ between (b) and (c) which we call rotational inconsistency.

Having detected an eye region, we now move to the problem of extracting the eye region. For distant-videos from the MBGC dataset, the IOM system acquires a face-visible video while a person walks through a portal at normal walking speed [10]. Unlike classic-still images obtained under rigid conditions, distant-video images have the characteristic that the subject’s head is not always straight to the camera. Apart from angle issues, the subject in a video might also blink or might have only one eye visible on the screen. In addition, a person can have a tilted head with various movements, which leads to a rotational inconsistency between two iris biometric samples. Rotational inconsistency means that the template starting points between two normalized iris image samples have a degree of distance difference caused by the head position as shown in Fig. 6.

**Fig. 7:**
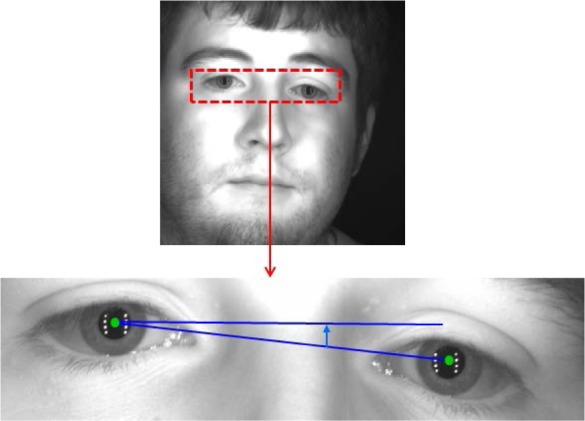
Angular alignment process using the left and right pupil information for an eye-pair image.

The horizontal dotted line (white) in Fig. 6 (a) is the starting point radius used for normalization, with a person’s eye looking straight at the camera. The horizontal solid line (yellow) in Fig. 6 (b) and (c) is the starting point radius of a person’s eye when the head was tilted. The white dotted lines in Fig. 6 (b) and (c) indicate the same location of the starting point from Fig. 6 (a)—as a person tilts his/her head, the white dotted line will also tilt. The larger angular difference between white (dotted) and yellow (solid) line indicates a greater rotational inconsistency that must be adjusted for achieving normalized images. We develop a method of image preprocessing before extracting the eye region and the method is illustrated in Fig. 7.

The center position of the pupil for the left and right eye is automatically detected from the eye-pair image (see details in Sec. 5.1). Using this pupil information, the positions of the left and right eyes are then angularly aligned according to the degree of the angular difference between the left and right pupil centers relative to the horizontal.

**Fig. 8:**
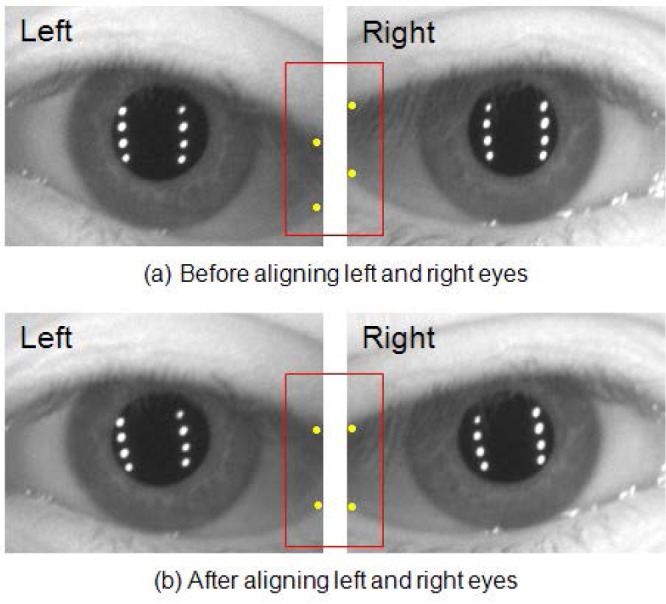
Eye position angular alignment process using the pupil center information; the red boxes show different locations of eyelids between before and after applying the eye position alignment method.

Figure 8 demonstrates the effect of this preprocessing step. The red boxes highlight the repositioning of the eyelids after the developed method has been applied.

The nose bridge region between the left and right eyes is eliminated with a scaled factor. In practice, the above-described algorithm works well, yielding a set of angularly aligned images that are then automatically saved (in BMP-format) into left-eye-only and right-eye-only files for further processing. For evaluation, we used 586 distant-videos that contain the eye-pair in at least one frame.

As illustrated in Table 4, a total of 4,796 eye-pair images were automatically extracted and saved in BMP format out of 11,341 frames (586 videos). The remainder of the 6,545 frames was either no eyes, only one eye visible, or totally black on the screen. The false positive rate was about 0.4 %.

### 4.3 Automated Image Quality Measurement (AIQM)

Most commercial iris recognition systems use images acquired under strictly constrained conditions with an integrated image quality control system. Near-infrared (NIR) illumination is normally used to light the face, and the user is prompted with visual and/or auditory feedback to correct eye position and distance. Consequently, the iris image is mostly in-focus and of sufficient resolution to facilitate iris recognition. In contrast, the face-visible videos captured under less-constrained acquisition conditions generally contain poorer quality images due to motion blur, being out-of-focus, poor illumination, glare (or reflections), blinking eyes, and other artifacts.

There have been several efforts to address dealing with poor quality images [20–22]; such methods include image quality assessment, image quality enhancement, and image restoration, among others.

The first approach (image quality assessment) is important because it gives an indication of:

1. whether an image is usable and recognizable
2. whether/how an image can be enhanced and restored
3. how an image can be compressed
4. the predictive performance of the matching results
5. which image out of a set of frames in a video sequence has the best quality (third sub-component of VASIR’s Video Processing module).

In the context of VASIR, we thus chose to focus our efforts on image quality assessment methods. Under generally less-constrained distant-video conditions, the ability to measure the eye/iris image quality in video streams provides valuable additional information which will yield more accurate matching results by determining which iris sample should go through the matching process or when the iris samples in video should be rejected altogether.

Several groups [10] have studied how to determine the focus of an image, e.g. analyzing the sharpness of the pupil and iris boundary, calculating the total high frequency power in the 2D Fourier spectrum, a variation of the Sum Modulus Difference (SMD) filter, etc. Spatial domain filters [22,23] are a direct way to capture image texture properties. This method measures the focus by calculating the edge density per unit area. Fine textures (in-focus) tend to have a higher density of edges per unit area than do coarser textures (out-of-focus). The measurement of an edge is normally computed over an image area by calculating a magnitude from the responses of the filters.

**Fig. 9:**
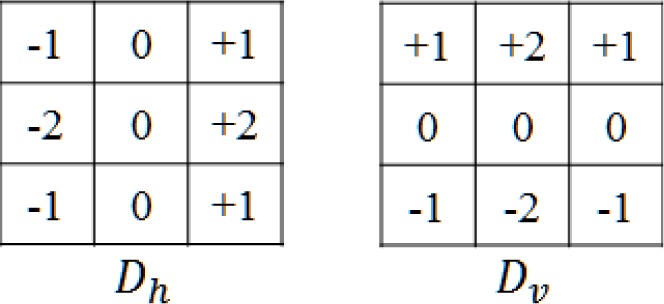
Sobel filters.

As a first attempt, the quality score was calculated by means of the edge density—as defined by the Sobel metric (see Fig. 9)—proposed for predicting the *face image* quality by Beveridge *et al.* [24]. The Sobel operator has been used extensively for image edge detection [22,25].

The Sobel operator consists of a pair of 3×3 filters—defined as D_h_ and D_v_ in Fig. 9. These filters are designed to respond to edges running horizontally and vertically relative to the pixel grid. The D_h_ and *D_ν_* are separately convolved with the input image *I*(*x*,*y*) to measure the gradient component in each orientation. The magnitude of the gradient *G* at each point is measured by:

(1) $$G(x,y)=\sqrt{{\left(D_{h}*I(x,y)\right)}^{2}+{\left(D_{v}*I(x,y)\right)}^{2}}$$

(2) $$SOB=\frac{\sum {}_{1}^{N}G(x,y)}{N}$$

where *N* is the total number of input image pixels and the *SOB* value is calculated by averaging the gradient over the entire image.

We applied the *SOB* metric to determine if the level of quality (mainly focus) can be predicted for *iris image* quality as well. We first calculated the quality scores using the *SOB* metric for all images from a set of query (*q*) and a set of target (*t*) iris images—also known as Gallery and Probe images respectively. The calculated quality scores were ordered to arrive at a final categorization of the quality level of specific images.

The Quality Score *(QS)* for a pair of images *q* and t—see details in [26,27]—is defined as,

(3) QS(q,t)=min{QS(q),QS(t)}.

Thus to calculate a pairwise quality score *QS*(*q*,*t*), the poorer quality score of the query image *QS*(*q*) and the target image *QS*(*t*) is chosen for a set of match (genuine) scores *M* and a set of non-match (imposter) scores *N*. The decision threshold *λ* and the image quality threshold *τ* must also be specified.

Therefore, the Verification Rate *V*(*λ*,*τ*)—also known as the True Accept Rate *TAR*(*λ*,*τ*)—is computed as follows:

(4) $$VR(\lambda ,\tau )=\frac{\#\{M\cap SS(q,t)\geq \lambda \cap QS(q,t)\geq \tau \}}{\#\{M\cap QS(q,t)\geq \tau \}}$$

where *M* is the set of match scores and *SS*(*q*,*t*) is the similarity score (e.g., Hamming distance) when the two images *q* and *t* are of the same person. The VR is a ratio, where the numerator is the number of similarity scores *SS*(*q*,*t*) greater than or equal to a decision threshold *λ*, and the denominator is the number of genuine comparisons for which the quality scores *QS*(*q*,*t*) is greater than or equal to a threshold *τ*.

The False Accept Rate *FAR*(*λ*,*τ*) is also of similarity measure and is computed as follows:

(5) $$FAR(\lambda ,\tau )=\frac{\#\{N\cap SS(q,t)\geq \lambda \cap QS(q,t)\geq \tau \}}{\#\{N\cap QS(q,t)\geq \tau \}}$$

where N is the set of non-match scores and *SS*(*q*,*t*) is the similarity score when the two images *q* and *t* are of different persons. The *FAR* is the number of similarity scores *SS*(*q*,*t*) greater than or equal to a decision threshold *λ*, relative to the number of quality scores *QS*(*q*,*t*) greater than or equal to a threshold *τ*.

**Fig. 10:**
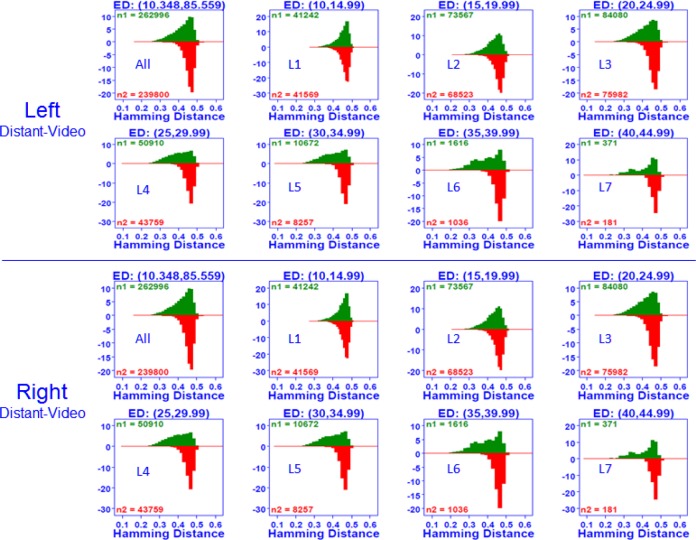
Bihistogram analysis of the quality score level—as defined by the Edge Density (ED) obtained from *Q(q,t)* using the Sobel filter—and Hamming distance for MBGCv2 *Left* and *Right* (green=matching (genuine) distribution, red=non-matching (imposter) distribution).

For analyzing behaviors of matching and non-matching distributions with quality scores, the quality scores were separated into seven groups by their quality level, ordered from low to high as presented in Fig. 10 for the left eye—note that the plot was generated with a NIST-developed analysis tool Dataplot [28,29].

A bihistogram [28,29] was used to plot the matching distribution (green) and the non-matching distribution (red) for each quality level. The x-axis is the Hamming Distance (HD) value and the y-axis is the frequency distribution of (Sobel) edge density scores. The Edge Density (ED) indicates the range of quality scores calculated by the Sobel metric (*n1* is the number of matching scores and *n2* is the number of non-matching scores).

The top-left-corner bihistogram in Fig. 10 presents the matching and non-matching distributions for the complete range of quality scores. The ED score for the MBGCv2 distant-video (left and right) dataset ranged from 10 to 86. Each of following seven plots (for left eye) presents the distribution of ED scores for subsequent subsets:

Level 1: ED (10, 14.99),
Level 2: ED (15, 19.99),
Level 3: ED (20, 24.99),
Level 4: ED (25, 29.99),
Level 5: ED (30, 34.99),
Level 6: ED (35, 39.99),
Level 7: ED (40, 44.99).

Note that we present only seven ED levels because only a small number of images have a quality score 45 or above. The amount of overlap between the matching and non-matching distributions indicates how well the genuine and imposter cases are classified; much overlap means poor classification; less overlap means better classification. The set of bihistograms in Fig. 10 thus allows us to display the behavior of the HD decision distribution between matching and non-matching scores.

Level 1 (ED:10,14.99)—see top-second plot in Fig. 10—has the largest overlap that is, has the most elements in common. The overlap area continues to decrease as the level number increases. We thus conclude that when the Sobel ED score is low we have poor quality images and hence have poor matching/non-matching classification results—so VASIR performs relatively poorly. On the other hand, when the Sobel ED score is high—see the row 2, bottom-last plot (Level 7) in Fig. 10—the overlap is much less than the Level 1 plot, hence the classification and VASIR performance is better than with the lower ED scores.

Because of increasing classification with increasing quality (as shown in Fig. 10), we conclude that the Sobel ED score serves as a good predictor of VASIR performance. Conversely, if we pre-measure the image quality and get a low ED score (e.g. Level 1 [10, 14.99]); this implies that carrying out the VASIR matching process in such cases may not be worth it. Further, we observe that while the matching (green) scores change shape and location from one plot to the next, the shape and location of the non-matching (red) scores is relatively consistent over different quality levels. Based on this, we arrive at the important conclusion that the main driver of the classification is not the non-matching score—but rather the matching score.

### 4.4 Best Quality Image Selection

This section addresses the question of how to select the best quality frame out of all frames within a face-visible video sequence involving a single person.

The first sub-section defines VASIR’s automated method for best-image determination called Automated Best Image Selection (ABIS). For instance, the system detects a number of left and right iris frames (sub-images) from a distant-video and then out of these detected iris frames—the system selects the best quality iris frame by using an automated quantitative quality measurement (AIQM) algorithm. As a benchmark, the second sub-section presents the results of a comparative experiment that was carried out involving VASIR’s automated approach (ABIS) versus a Human-based Best Image Selection (HBIS).

#### A. Automated Best Image Selection (ABIS)

**Fig. 11:**
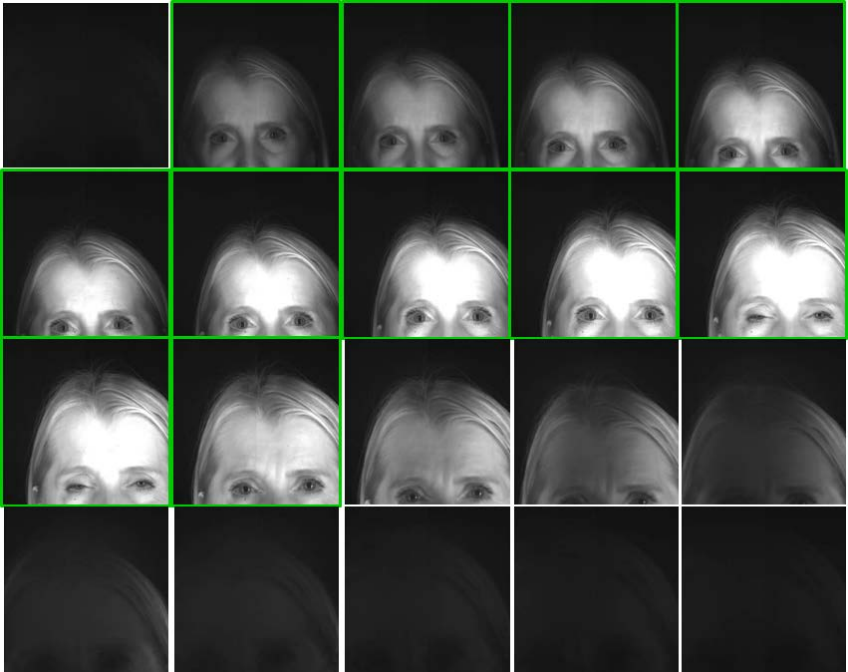
All 20 frames from a representative video sequence in the MBGC distant-video dataset (2024×2024 pixels resolution).

One method for determining the best frame is to compute the quality score based on the Sobel metric for all frames in the sequence and choose the frame with the highest quality score. The pictures in Fig. 11 show an example of the best image selection process for a video stream; this figure shows all 20 frames from the original video sequence.

Note that some of the frames do not include an eye-pair, or contain only part of the eye region.

**Fig. 12:**
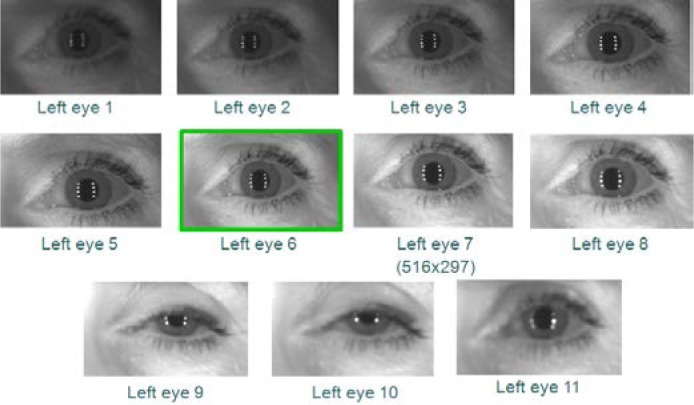
Left eye: the best quality image selected by the *ABIS* method was frame 6.

**Fig. 13:**
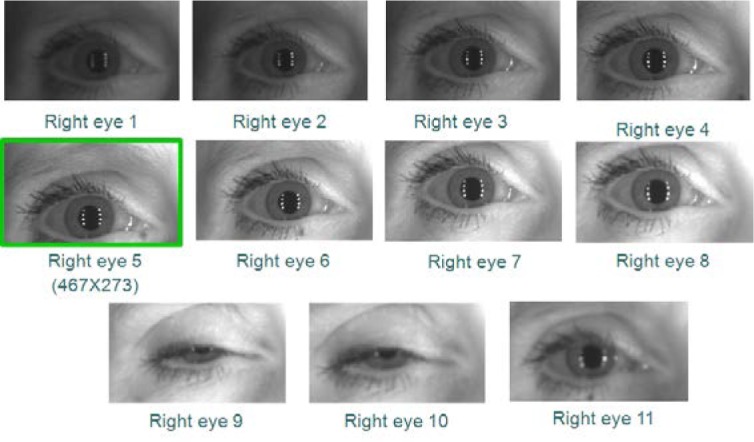
Right eye: the best quality image selected by the *ABIS* method was frame 5.

However, using VASIR’s eye region detection/extraction method (as described in Secs. 4.1 and 4.2), 11 frames for each left and right eye were (automatically) detected/extracted out of a total 20 frames to yield the subset shown in Fig. 12 (left eye) and Fig. 13 (right eye).

These extracted iris images are passed to the automated best image selection (ABIS) step to calculate the quality score and choose the best frame with the highest quality score. The detailed ABIS algorithm is as follows:

1. Load a frame F_i_
2. Detect the eye-pair in F_i_ and extract the left and right iris images separately
3. Calculate the quality score using AIQM (Automated Image Quality Measurment)
4. Choose the frame with the highest value by comparing the previously highest and current quality score
5. Repeat steps (1) to (4) until F_n_
6. Save the frame with the highest quality score.

As an example, out of these 11 images in Fig. 12 and Fig. 13, the 6^th^ frame (516×297 pixels) was selected as the best quality image for the left eye and the 5^th^ frame (467×273 pixels) for the right eye—note that the extracted iris image resolution varies due to the subject movement and distance.

#### B. Human Best Image Selection (HBIS)

**Fig. 14:**
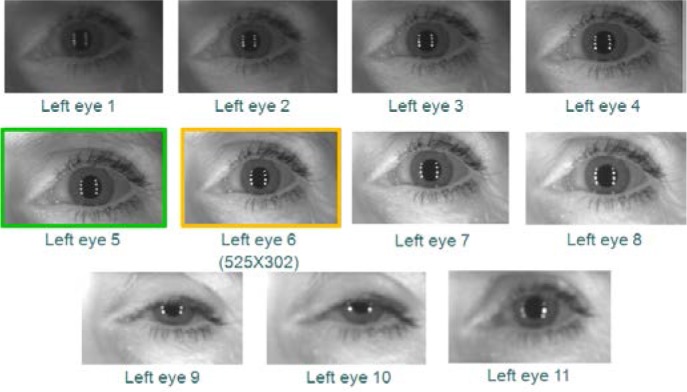
Left eye: the best quality image selected by the *HBIS* method was frame 5.

**Fig. 15:**
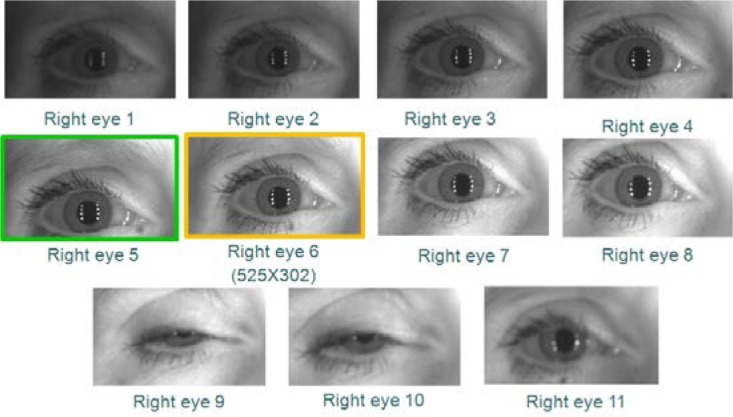
Right eye: the best quality image selected by the *HBIS* method was frame 5.

As a point of reference, we carried out (on the same image data) a parallel human vision evaluation to select the best quality image as ground truth and compared it to the ABIS results. This evaluation involved two people and the MBGC distant-video datasets. The ground truth protocol was defined as follows:

1. We supplied two persons with all iris images extracted from all MBGC video sequences. The left and the right iris images were given separately—as shown examples in Fig. 14 and Fig. 15.
2. The associates were instructed to consider images that contained a closed eye, were too dark/bright, had a partially occluded iris region, or were out-of-focus to be of low quality and to be set aside.
3. Based on this awareness, the two people were asked to pick the two images that they considered to be the best image and second best quality image for each sequence. We decided beforehand to allow each person to pick two frames instead of one due to the practical difficulty of distinguishing between the top quality images via human vision—the human visual system is not quantitative.
4. To strengthen the final decision as to which pair was the best, we segmented the pupil boundary, iris boundary, and the eyelid curves to isolate the iris region from the two selected images. Since the segmentation stage is known to have a high influence on iris recognition performance, we used the segmentation results as a tiebreaker for distinguishing the best from the next-best.
5. Based on the precision of the segmentation result, we finally selected the best single frame. In case both iris segmentations were successful or both failed, we selected the first picked best image by default.

For the same image data used in the ABIS results of Fig. 12 and Fig. 13, we show the corresponding HBIS results in Fig. 14 (left eye) and Fig. 15 (right eye).

For the left eye, the 5^th^ and 6^th^ frames were selected via human vision, and then the 5^th^ frame (525×302) was selected as the best frame from the successive segmentation result. For the right eye, the 5^th^ frame (525×302) was also selected as best using the HBIS method.

**Fig. 16:**
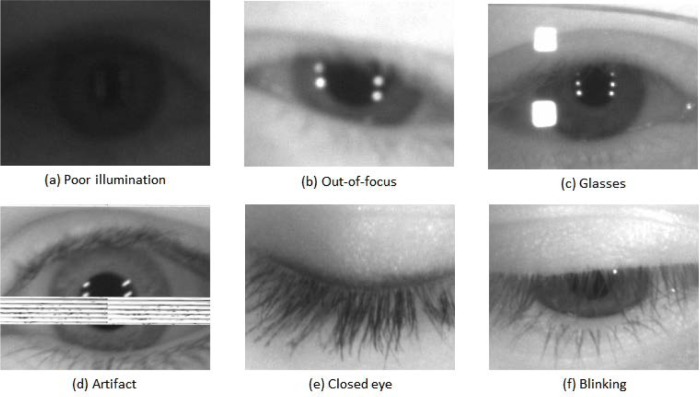
Examples of poor quality images even though these were selected as best quality image by the human vision (HBIS)—from MBGC distant-video dataset.

Note that all 586 video sequences have a best image—but not all best images are of optimal quality images per se. Figure 16 is a collection of the six problematic best images using human vision (HBIS) from six (out of the 586) video sequences in the MBGC distant-video dataset.

All frames of these six video sequences were uniformly of poor quality in some respect and so the resulting six *best images* were still poor. These examples illustrate the level of challenges we encountered in our study.

**Fig. 17:**
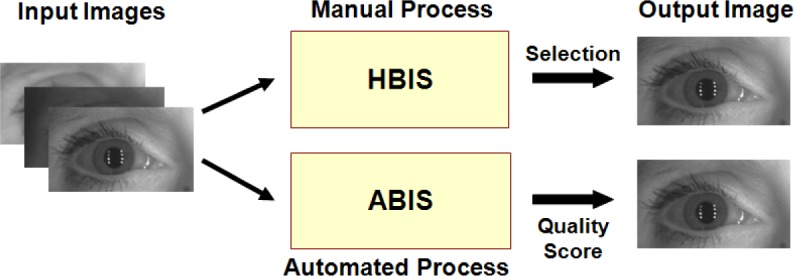
Automated method (ABIS) is compared to ground truth (HBIS).

As a next step, the method developed for image quality assessment was examined to determine how well the automated quality assessment algorithm (ABIS) correlated with the human assessment of quality (HBIS)—note that human vision is subject to error in itself and it should not be treated as ultimate ground truth. Figure 17 shows the comparison procedure of ABIS and HBIS.

Using the MBGC dataset, we compared ABIS and HBIS approaches by evaluating the resulting VASIR verification rate (VR).

Out of the 586 videos, two sub-datasets were extracted as follows:

1. The first set of 586 (×2=1,162 best frames for left and right) was selected out of these 586 videos using the ABIS approach.
2. The second set of 586 (×2=1,162 best frames for left and right) was selected by the HBIS approach.

**Fig. 18:**
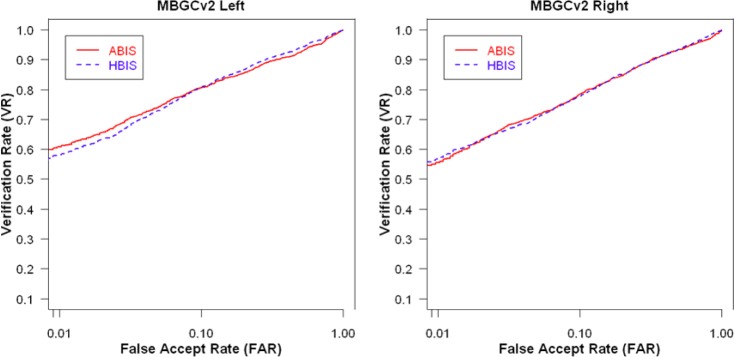
Comparison of ABIS and HBIS for VASIR performance from MBGCv2 Left/Right distant-video.

Figure 18 shows the VASIR verification performance results from ABIS and HBIS plotted using ROC curves.

The x-axis is the False Accept Rate (FAR) and the y-axis is the Verification Rate (VR). The solid curve indicates the results from the ABIS method and the dotted curve indicates the results from the HBIS method. The plots show that the automated algorithm (ABIS) is nearly equivalent to ground truth using the human vision (HBIS).

This thus suggests that VASIR is not only near-unique in that its algorithm automatically extracts the best image from a video sequence, but also automated best image selection (ABIS) of VASIR is almost as good as its (manual) human ground truth counterpart for the MBGC dataset.

## 5. The VASIR Iris Recognition Module

Iris recognition has been playing an important role in the biometric community due to its high accuracy and stability. From a user standpoint, however, iris recognition systems are frequently difficult to interact with. The positioning of the eye and the time taken for the enrollment process put strains and demands upon the user. This leads to the question, as to why do iris-based biometrics need to be so rigorously stringent in regards to their image acquisition procedure? The answer is that iris recognition normally takes place in multiple stages, whereby each stage is potentially very sensitive depending on the algorithm used to the present image quality conditions and noise. We developed the VASIR system to be more resistant to such noise and varying image quality conditions.

To this end, we developed a new approach for the iris segmentation and optimized the normalization resolution. A new noise-masking scheme for feature extraction and encoding is also introduced. Along with this work, a new bit shifting method for comparing the similarity of two images is presented that aims to reduce rotational inconsistency and segmentation inaccuracy. The Iris Recognition module consists of four components: segmentation, normalization, feature extraction/encoding, and similarity distance metric.

### 5.1 Segmentation

Iris segmentation can be a challenge especially for images with partial occlusions due to noise, motion blur, subject movement, poor illumination, artifacts and distortion, among other defects. Therefore, VASIR introduces a new iris segmentation methodology which uses a combination of contour processing and Hough transform algorithms—along with a new approach to eyelid detection.

#### A. Pupil Boundary Detection

User-specification is reduced by using automatic threshold selection to detect the pupil and by using the pupil radius to be the minimum radius of the iris. The pupil region is normally of low intensity in the eye image—hence we first look for the smallest intensity value within the eye region image. To find the optimal threshold value for detecting the pupil boundary automatically, VASIR proposes to measure contrast within an image based on the GLCM (Gray Level Co-occurrence Matrix) method [30,31]—also known as the Gray Level Spatial Dependence method. Contrast is a measure of the intensity differences between a pixel and its neighbor over the whole image. The GLCM approach uses a two dimensional histogram of gray levels for all-possible pairs of pixels, which is computed using a displacement vector, defined by its radius *d* and orientation *θ*. The matrix element *P*(*i*,*j*|d,*θ*) contains the second order statistical probability values for changes between gray levels *i* and *j* at a particular displacement distance d and at a particular angle *θ*—see details in Albregtsen [30]. To avoid direction dependency, an average matrix out of four matrices, *θ*={0°,45°,90°,135°} can be calculated as the given angular mean, *M_T_*(*d*):

(6) $$M_{T}(d)=\frac{1}{N_{\theta }}\sum_{\theta }T(d,\theta )$$

where *T*(*d,θ*) is the number of scalar texture measures and *N_θ_* is the number of angular measurements.

Based on this, the following contrast metric can be derived:

(7) $$CON=\sum_{i=0}^{G-1}\sum_{j=0}^{G-1}{\left(i-j\right)}^{2}P(i,j)$$

where *G* is the number of gray levels. A higher contrast is considered as to be an indicator of better quality in our study. Hence, the final threshold *T* is defined:

(8) $$T=\left(\frac{\mathrm{CON}}{Norm}+1.0\right)\times (\min_{i,j}\{I(x_{i},y_{i})\}+\alpha )$$

where *I*(*x_i_,y_j_*) is the eye region image and *α* is an additive constant for defining the threshold. Empirically, we noted that a norm of 256 yielded superior matching resulting and so was used as the norm value in our study.

If no pupil boundary is found, then the threshold value is automatically increased or decreased using the value of *α* until the pupil boundary is found. VASIR’s method for detecting the pupil boundary applies contour processing without the need for a user-given threshold value, a minimum size for the circles, or a starting point. The contour processing proposed by Teh-Chin [32] searches for contour points in a shape of interest, utilizing the method of maximal local differential within an image. The pupil segmentation method proposed by Li *et al.* [33] detects the pupil boundary by applying ellipse fitting to the contour points. Li’s algorithm requires the user to specify a threshold value and to locate a starting point to find the pupil center. VASIR overcomes this by applying a simple thresholding method to find the pupil center point automatically. The result *T* from Eq. (7) is then used as the threshold value to detect the pupil boundary. To detect the pupil boundary as fast as possible, binary thresholding [22] is applied.

**Fig. 19:**
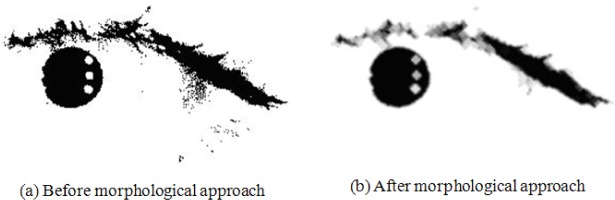
Example of noise reduction with morphological technique in VASIR.

The presence of noise in our dataset could be caused by blurriness, eyelashes, eyelids, glasses, reflections, lens distortion, or shadows; the Teh-Chin algorithm does not, however, perform well in such noisy environments [34]. To reduce the noise in an image, VASIR applies a Gaussian filter to the binary threshold image and uses morphological opening and closing later on. *Opening* of an image is an erosion followed by a dilation to remove small objects from a binary image while preserving the shape and size of larger objects within the image. *Closing*, on the other hand, consists of dilation followed by an erosion to eliminate small holes and fill gaps in the image [22]. The process of finding an optimal threshold value this way is possibly slower since the optimal threshold is calculated differently depending on the amount of noise within an image. However, we found this method to be much more effective for reducing noise as shown in Fig. 19.

Following the noise removal, our algorithm searches for the largest local quantity of contour points, i.e. it searches for the biggest circle within the image. This assists greatly in ignoring the noise from the eyelashes, eyebrows, and other artifacts.

To find the circle of the pupil, we employ the general elliptical fitting method suggested by Fitzgibbon *et al.* [35]. In his paper, a general conic is represented by:

(9) $$F(a,x)=a\cdot x=ax^{2}+bxy+cy^{2}+dx+ey+f=0$$

where *a*=[*a*,*b*,*c*,*d*,*e*,*f*]*^T^* and *x*=[*x*^2^,*xy*,*y*^2^,*x*,*y*,1]*^T^*.

*F*(*a*,*x_i_*) is called the “algebraic distance” of point (*x*,*y*) to the conic *F*(*a*,*x*)=0. Minimizing the sum of squared algebraic distances of the curve to the *N*th data point *x_i_* can be expressed as:

(10) $$D_{A}(a)=\sum_{i=1}^{N}F{\left(a,x_{i}\right)}^{2}=||Da|{|}^{2}$$

To fit ellipses, the discriminate b^2^−4ac should be negative. However, Fitzgibbon’s method imposes the further equality constraint 4*ac*−*b*^2^=1 to incorporate scaling into the constraint. The constrained ellipse fitting problem reduces to minimizing ||*Da*||^2^, where the design matrix D=[x_1_,x_2_,…,x_n_]^T^ subject to the constraint *a^T^Ca*=1; *C* is a 6×6 constraint matrix. The system can be expressed using the equation below by introducing the Lagrange multiplier *λ* and differentiating;

(11) $$2D^{T}Da-2\lambda Ca=0,\;a^{T}Ca=1$$

which may be rewritten as;

(12) $$Sa=\lambda Ca,\;a^{T}Ca=1$$

where *S* is the scatter matrix *D^T^D*. Based on the Fitzgibbon’s approach, we derive the center and size of the ellipse in VASIR.

**Fig. 20:**
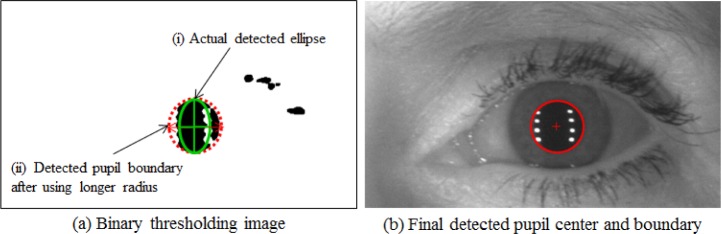
Pupil boundary detection with the ellipse fitting method; Left image: note that (a) actual detected ellipse (b) detected pupil boundary after using longer radius. Right image: original image with the superimposed pupil boundary.

Note however that reflections—also known as highlights—can cause information loss which leads to a potentially incorrect pupil boundary. While it seems possible to detect all reflections and to interpolate each reflection region with its neighbor pixels, our studies have shown that this simply takes too long to be a realistic approach. To simplify processing, we assume that the pupil’s shape is a perfect circle. Under this assumption, we select the radius of the pupil’s circle based on the length of the major ellipse axis. The procedure of our pupil boundary detection is visualized in Fig. 20.

The green solid ellipse in Fig. 20 (a) (i) is the actual ellipse detected using the fitting method, while the red dotted circle in Fig. 20 (a) (ii) is the pupil circle that was determined by choosing the larger value between the width and height of the ellipse as the circle radius. Figure 20 (b) presents the final outcome of the pupil boundary segmentation. Recent work from Li’s pupil detection [36] has also shown the effects of selecting the major axis to detect the pupil circle. VASIR’s pupil detection method is less sensitive than Li’s *et al.* algorithm [33] to image variations, such as eyelashes accentuated by heavy mascara, and the pupil occlusions due to reflections.

#### B. Iris Boundary Detection

The iris boundary is more difficult to distinguish than the pupil boundary because of the lower intensity difference between iris boundary and sclera region—especially for poor contrast images. The Hough transform is very useful for working with images that include noise and are partially occluded [37]. More than 45 % of the iris images that were extracted from the MBGC NIR face-visible video dataset are corrupted with noise, are out-of-focus, and have poor contrast. For those images it is better to use the Hough transform to detect the iris boundary rather than contour processing—see details in Duta *et al.* [38] and Pedersen [39].

This particular iris segmentation algorithm is based on a *circular* Hough transform as initially employed by Wildes *et al.* [40]. We used the circular Hough transform algorithm implemented by IrisBEE for the VASIR system.

As mentioned before, IrisBEE’s Hough transform based segmentation algorithm needs a custom value tailored to each dataset. VASIR overcomes this dependency by using the detected pupil region as the lower limit for determining the iris circle radius. We set the upper limit based on the scale factor of our system. It is commonly known that while the iris and the pupil centers are not concentric [41,42], their centers are only slightly divergent; hence, VASIR uses the center coordinates of the pupil to reduce the number of false positives when trying to detect the iris boundary. As a consequence, some false positives get introduced when VASIR fails to correctly detect the pupil center; however, such occurrences are sufficiently infrequent that using the pupil center still provides a significant advantage. The average false positive rate by use of the pupil center is reduced to approximately 1.4 %.

#### C. Eyelids Detection

**Fig. 21:**
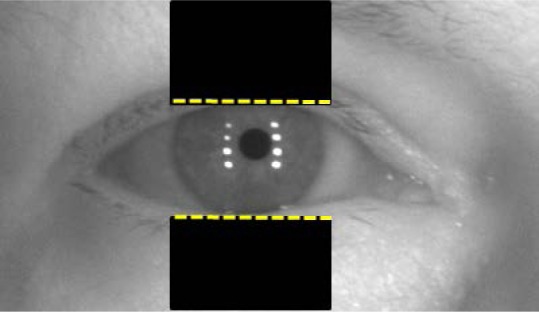
Eyelid horizontal line detection using IrisBEE’s algorithm.

The eyelid detection in video imagery turned out to be an especially challenging task. As mentioned above, in IrisBEE’s algorithm, the eyelids were removed using a *linear* Hough transform to replace the upper and lower eyelids with horizontal lines. Figure 21 below illustrates an example of the eyelid detection using IrisBEE’s algorithm.

The black rectangles in Fig. 21 assist in eyelid detection by inserting horizontal flat lines to delimit the upper and lower eyelids—see highlights in yellow. However, the insertion of the horizontal flat lines are not an optimal solution for detecting eyelids, because eyelids are not per se straight, and/or a person’s head might be tilted or rotated off-horizontal. It is important also to understand that human eyes are known to have different curves for the upper and lower eyelids. Further, the shape of the eye can be significantly different depending on the person (e.g., race: Caucasian, Asian, etc.).

**Fig. 22:**
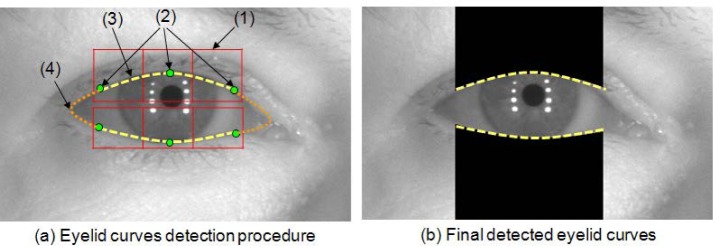
VASIR’s eyelid curves detection using linear Hough transform and Lagrange interpolation; (a) shows each step for detecting eyelid curves, (b) shows the final segmentation for upper and lower eyelids.

In this study, two different curves are used to segment the upper and lower eyelid curves as shown in Fig. 22.

VASIR’s eyelids detection method has four steps as described below:

1. The determination procedure starts with splitting the upper eyelid region of the eye image into three parts (see Fig. 22 (a):(1)).
2. The three points on the upper eyelid are detected by a linear Hough transform (see Fig. 22 (a): (2)). A linear Hough transform involves transforming each of the points to a parameter space to search for a straight line. Every point in the *x-y* space can be transformed to the *a-b* parameter space as given by this equation:
  (13) $$b=-x_{i}a+y_{i}.$$
  This representation has, however, the problem that the value of can be infinite for vertical lines. An alternative representation is to specify a line by the angle *θ* of its normal and its algebraic distance *r* from the origin:
  (14) $$r=x_{i}\cos\theta +y_{i}\sin\theta ,$$
  where *θ* is within [0, π] and *r*≠0 Both *θ* and *r* are constant for each of the points (*x_i_*,*y_i_*) on a straight line.
3. Lagrange interpolation is applied to the curve (see Fig. 22 (a): (3)). The Lagrange formula for interpolating between points can be formed as follows: Given a finite number of points *x*_1_,…*x_N_*, several real constants *y*_1_,…,*y_N_* and a subspace *V* of *∏^d^* (where *V* is the interpolation space and *∏^d^* is the space of all d-variant polynomials), finds a polynomial *p*∈*V*, such that,
  (15) $$p(x_{j})=y_{j},\;j=1,\ldots ,N.$$
  (16) $$p(x)=\sum_{i=1}^{N}y_{i}\ell_{i}(x),$$
  where
  (17) $$\ell_{i}(x)=\prod_{\begin{array}{c} j=1\\ j\neq i \end{array}}^{N}\frac{x-x_{j}}{x_{i}-x_{j}},\;i=1,\ldots N,$$
4. The same procedure of steps (1) to (3) is repeated to detect the lower eyelid curve as well.

This Lagrange interpolation method works very well in practice. Figure 22 (b) shows the final result of the eyelid curves segmentation. The result indicates that the upper and lower eyelid curves follow well the actual eyelid shape of the individual.

#### D. Segmentation Results

The segmentation performance of VASIR was evaluated using the MBGCv1 distant-video dataset. For this dataset, there are a total of 149 video sequences for NIR face-visible video collected from 114 subjects by the IOM system. Of the 149 sequences, only 126 videos are usable because 23 video sequences had either no eye visible or one eye visible. Like VASIR, IrisBEE is a non-commercial and open-source system, and so the segmentation results of VASIR were compared to IrisBEE.

For the 252 (= 126×2) eye images out of 126 videos, IrisBEE’s segmentation rate from the iris images that were selected by ABIS (Automated Best Image Selection) was 44.1 % for “total” detection: the pupil, iris, and the eyelid. The results for the iris images that were manually selected by HBIS (Human Best Image Selection) was 48.6 %.

**Fig. 23:**
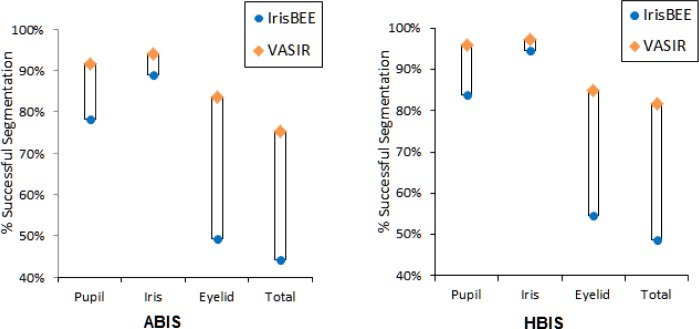
Comparison between VASIR and IrisBee’s segmentation algorithm using MBGCv1 distant-video.

Using the segmentation method developed for VASIR, the rate for “total” detection improved to 75.4 % for ABIS and 81.7 % for HBIS. Figure 23 is a comparison of VASIR and IrisBEE segmentation results.

**Fig. 24:**
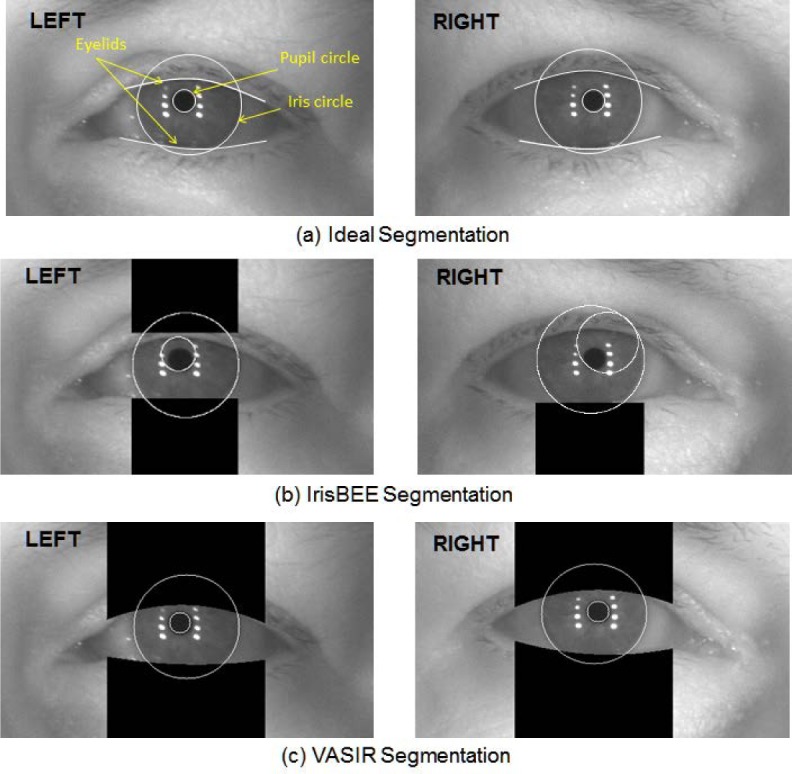
Comparison of (a) Ideal segmentation (b) IrisBEE’s approach and (c) VASIR’s approach.

The blue circles indicate the segmentation results for IrisBEE and the orange diamonds the ones for VASIR. Note that for each feature (pupil, iris, eyelid, and total) on the horizontal axis, the VASIR successful segmentation rate is uniformly higher than the IrisBEE success rate. For both ABIS (Automated Best Image Selection) and HBIS (Human Best Image Selection), VASIR has a total 31 % higher successful segmentation rate than IrisBEE. Particularly, significant is that the VASIR segmentation method leads to an enhancement (32.3 %) of the eyelid detection compared to IrisBEE’s algorithm. Figure 24 illustrates the improvement in segmentation quality between VASIR and IrisBEE.

The results indicate an advantage of our method that the upper and lower eyelid curves follow the actual eyelid shape of the individual. The overall results show that the VASIR’s segmentation approach is superior to IrisBEE’s approach.

### 5.2 Normalization

VASIR—as an iris recognition system intrinsically involves the comparison of two biometric iris samples. Even for multiple images of the same subject, a complication arises in such a comparison due to pupil dilation, non-concentric pupil displacement, and/or varying eye distance to the capture device. To facilitate the comparison, these multiple images must be stretched or compressed to a standardized scale (normalization).

For the normalization step, a standardized 2*D* image of the iris region is generated by a polar coordinate-based method [42] based on two circular constraints (pupil and iris). The method accommodates issues caused by the above-mentioned complications and effectively eliminates scaling problems caused by linear stretching.

**Fig. 25:**
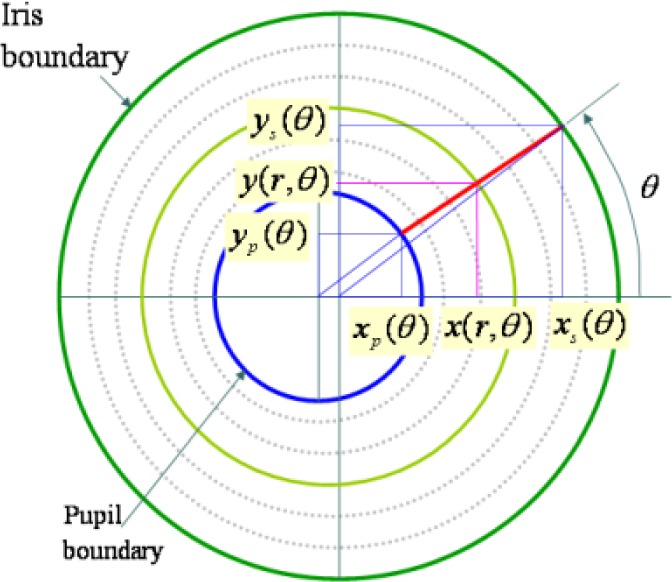
Projected polar coordinate system—image modified from the presentation [43].

The rubber sheet model as shown in Fig. 25 assigns to each point within the iris region a pair of real coordinates (*r*,*θ*) where the radius *r* lies on the unit interval [0,1] and *θ* is the angle over [0,2*π*]. The remapping of the iris image *I*(*x*,*y*) from Cartesian coordinates (*x*,*y*) to polar coordinates (*r*,*θ*) is classically represented as:

$$\begin{array}{c} I\left(x(r,\theta ),y(r,\theta )\right)\to I(r,\theta )\\ x(r,\theta )=(1-r)x_{p}(\theta )+rx_{s}(\theta )\\ y(r,\theta )=(1-r)y_{p}(\theta )+ry_{s}(\theta ) \end{array}$$

where $\left(x_{p}(\theta ),y_{p}(\theta )\right)$ and $\left(x_{s}(\theta ),y_{s}(\theta )\right)$ are the coordinates of the pupil and iris boundaries respectively along the *θ* direction.

As in IrisBEE’s algorithm, the pupil center is used as a reference point, and the iris region is scaled based on the radial vectors. Finally, an iris pattern image is generated by normalizing the angular size (240 pixels) and the radial size (20 pixels). A noise mask of the same size as the iris pattern image is generated using the occlusion information (e.g. eyelids, eyelashes, and reflections) obtained in the segmentation stage.

**Fig. 26:**
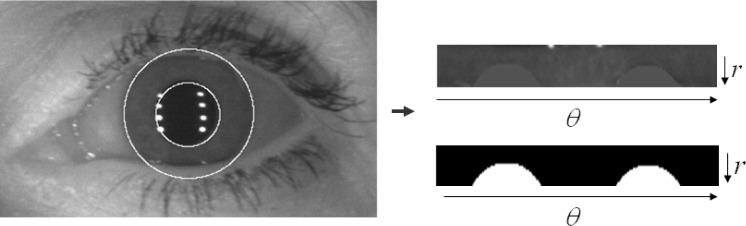
Normalized 2D iris pattern image and the mask that included the noise information; left image shows the original image with the pupil and iris circular information, the right-top image shows the normalized iris image, and the right-bottom is an example of the noise mask.

Figure 26 illustrates the normalized 2D iris pattern image and its noise mask that included occlusion information derived in the segmentation step. The left side in Fig. 26 is the original image with the pupil and iris circular information. The right-top is the normalized version of the iris region using the polar coordinate system, and the right-bottom is the normalized version augmented with the noise mask containing the occlusion information.

To find the optimal angular and radial resolutions, an optimization approach based on a 3^2^ full factorial design in *r* and *θ* was applied. Our results show that VASIR obtained a higher performance with a circumferential angular size of 260 pixels and a radial size of 34 pixels.

### 5.3 Feature Extraction/Encoding

For the feature extraction component, many different filters have been suggested in the literature [10] to represent the patterns from the iris region (e.g. Laplacian-of-Gaussian filter, Gabor-filter for wavelet transform, and discrete cosine transform).

The IrisBEE algorithm employed a 1D Log-Gabor filter—introduced by Yao *et al.* [44]—to process the feature extraction from normalized iris images. For this method, a Fast Fourier Transform (FFT) is first applied to the normalized iris image. The complex values from FFT are then convolved by filters that have Gaussian transfer functions when viewed on the logarithmic frequency scale. The frequency response of a Log-Gabor filter is given as:

$$G(w)=e^{-\log{\left(\frac{w}{w_{0}}\right)}^{2}/2\log{\left(\frac{\sigma }{w_{0}}\right)}^{2}}$$

where *w*_0_ represents the filter’s center frequency and *σ* gives the filter bandwidth. The complex values are then applied by an inverse Fast Fourier Transform (iFFT).

**Fig. 27:**
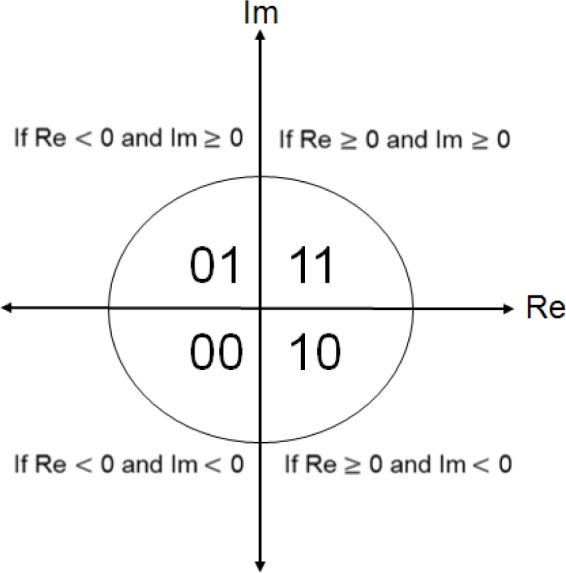
Encoding process with four quadrants using complex values.

To encode these (complex-valued) coefficients with the binary iris code [0, 1], the phase information with four quadrants proposed by Daugman [42] is employed (see Fig. 27). To determine each bit of the iris code for the coefficient, if the real part of the coefficient is negative, the iris code is mapped to “0”, otherwise it is mapped to “1”. If the imaginary part is negative, the iris code is mapped to “0”, otherwise it is mapped to “1”.

Further, Hollingsworth *et al.* [45] pointed out the existence of “fragile bits” in the generated iris code. The fragile bit theory declares that not all bits in the iris code are of equal consistency; coefficients in close proximity of the axes for both the real and imaginary can be a 0 for some images and a 1 for other images of the same iris. This concept gave birth to the idea that bits corresponding to complex filter responses *near the axes of the real or imaginary part* should not be included when measuring the similarity distance.

Hollingsworth’s approach looks at the real or imaginary parts separately to identify fragile bits. This approach can lead to incorrect results—e.g., loss of significant information of the iris pattern or incorrect classification as outliers—because a pixel within an iris image is represented by two bits of complex values, not a single bit. In other words, if an outlier exists in an image, the outlier should be ignored based on the complete information of the pixel, namely the joint information of the real and imaginary parts.

For the design of VASIR, we have explored and proposed a different masking scheme than that used by Hollingsworth’s approach. Instead of masking out fragile bits where the coefficient is *close to either the real axis or the imaginary axis*, we base our mask on *the distance (magnitude) of the coefficient from the origin*.

**Fig. 28:**
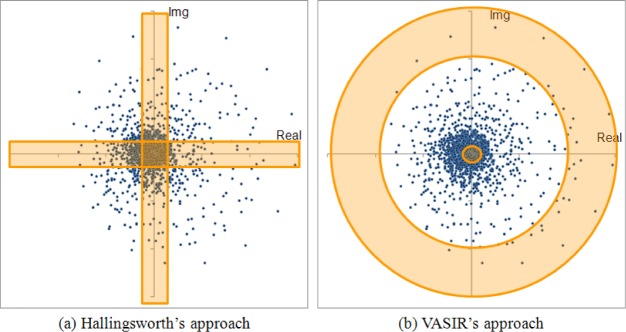
Comparison of (a) Hollingsworth’s approach and (b) VASIR’s approach—Scatter plot of raw wavelet coefficient from the normalized iris image using MBGC distant-video (video file name: 04327v1128).

Figure 28 shows a difference between the Hollingsworth’s approach and VASIR’s approach—using a Scatter plot of 4,800 points of raw wavelet coefficients from a normalized iris image (240 × 20 pixels)—plotted using one of frames from a video file in the MBGC dataset. For comparison purpose, we chose in Fig. 28 to use the same angular (240) and radial (20) resolutions as employed by Hollingsworth.

As illustrated with the color-shaded area in Fig. 28 (a), Hollingsworth’s approach masks out points based on fragile bits that are near to either the real axis or the imaginary axis—we call such points “mask-if-fragile”. In contrast to the Hollingsworth’s approach, our approach in VASIR (see the color-shaded part in Fig. 28 (b)) is to mask out points that are either very close or very far away from the origin—we call them “mask-if-close-and-far”. The idea behind VASIR’s approach is if the amplitude of wavelet is too small or too large, we assume that the pixel may be noise or an outlier, respectively.

**Fig. 29:**
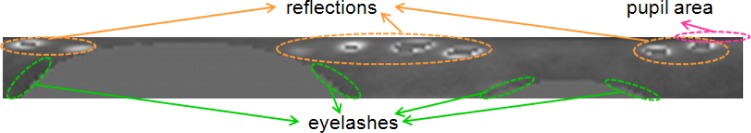
An original normalized iris image (baseline) with annotated information about eyelashes, reflections, and pupil regions.

Figure 29 is an original normalized iris image—which we call “baseline”—with annotated outlier information such as eyelashes, reflections, pupil regions, among others. Note that a good masking scheme should automatically highlight these regions for rejection.

**Fig. 30:**
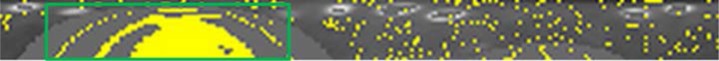
Mask-if-fragile: *Hollingsworth’s approach* of masking out the pixel of “fragile bits” where bits are near to either the real axis or the imaginary axis (masked out 10 % of fragile bits—see yellow area).

Figure 30 shows the effect of applying the “mask-if-fragile” scheme of Hollingsworth’s approach.

Figure 30 shows the normalized iris image with pixels marked in yellow color where bits were within 10 % of an axis. Most of those pixels were found to be within the area of the green rectangle—which will be masked out using the noise mask described in Sec. 5.2 (see Fig. 26). Consequently, the pixels within the green rectangle box should be ignored. Other pixels in yellow were found in various places within an image.

**Fig. 31:**
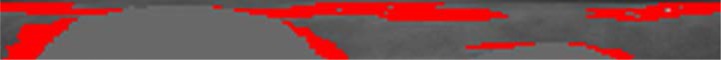
Mask-if-close-and-far: *VASIR’s approach* of masking out pixels with small magnitude values (a) masked the pixels with a magnitude below 1 % and above 80 % (see red area).

Figure 31 illustrates the effect of applying the VASIR masking scheme.

Figure 31 (a) shows the normalized iris image with pixels marked in red color whose magnitude value was *below* 1 % and *above* 80 %. Most of those pixels were found in the region near reflections (highlights), eyelashes, or the pupil area resulting from incorrect segmentation—all considered noise in an iris image. In this analysis, the “mask-if-close-and-far” VASIR approach is superior to the “mask-if-fragile” approach. In addition, this analysis shows that most of eyelash/reflection outliers have a large magnitude value rather than a small magnitude value.

For evaluation, we examine the question as to whether our developed feature extraction algorithm—using the VASIR’s masking scheme of the magnitude of wavelet coefficients—improves the VASIR performance compared to a baseline (no magnitude information used) and Hollingsworth’s fragile bits approach.

We used a 586-image subset (× 2 = 1,162 for left/right) out of all best frames from the MBGCv2 distant-video left/right dataset. We used two criteria:

1. Verification Rate (VR) at a fixed value (0.01) of the False Accept Rate (FAR)
2. EER (Equal Error Rate) value.

We picked VR at a FAR of 0.01 due to the small number of subjects within the subset.

We conducted a “baseline” experiment in which no magnitude-based masking was used. For Hollingsworth’s experiment, we masked out 10 % of fragile bits because the suggested quartile rate in her paper [45] resulted in a far worse performance. For VASIR’s “mask-if-close-and-far”, we have chosen below 1 % and above 80 % because these parameters were optimized via appropriated orthogonal experiment design methodology.

**Fig. 32:**
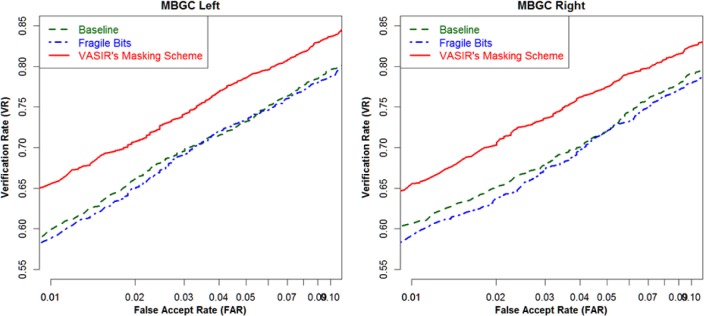
Comparison of Baseline, Hollingsworth’s approach (fragile bits), and VASIR’s approach for MBGC distant-video left/right.

Again, the use of the VASIR’s mask-if-close-and-far approach led to a significant enhancement of the VASIR performance compared to the baseline and the fragile bits approach—as presented in Fig. 32.

Our experiments show that the VASIR’s feature extraction approach is excellent for masking “outliers” correctly, which correspond to noise regions such as eyelashes, reflections, and pupil area (which were incorrectly localized from the segmentation stage).

### 5.4 Similarity Distance Metrics

Similarity metrics provide a quantitative evaluation of how similar the target and reference templates are. A wide variety of similarity metrics have been proposed in the iris-based biometrics community [40,41,46]. The “best” metric to be utilized in a particular application depends on the applied feature extraction algorithms, and on how the data is transformed and encoded. For instance, the Hamming distance (HD) metric is an appropriate measuring tool for the similarity measure of binary strings.

In VASIR, a fractional HD as initially proposed by Daugman [41] and later re-implemented by Masek [6] was applied to its iris recognition system. The fractional HD is given by:

$$HD(T,Q)=\frac{\sum_{i=1}^{N}\left(T_{i}⊕Q_{i}\right)\cap (Tm_{i}\cap Qm_{i})}{N-\sum_{k=1}^{N}\left(Tm_{k}\cup Qm_{k}\right)}$$

where *T* and *Q* are two bit-wise templates and *Tm* and *Qm* are the corresponding noise masks. These noise masks help to exclude the insignificant bits (e.g., eyelashes, eyelids, reflections, etc.) of a template. The total number of bits in a template is *N.*

**Fig. 33:**
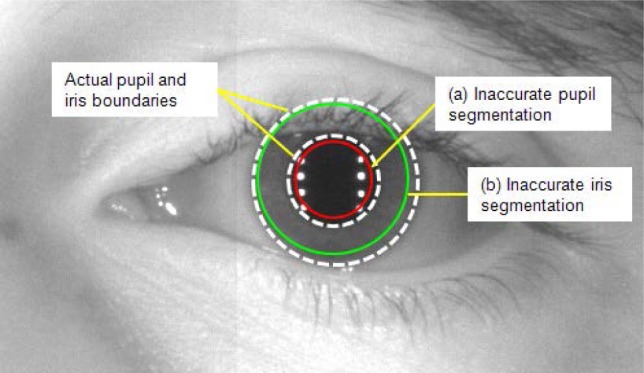
Example of inaccurate segmentation (a) pupil and (b) iris boundary.

The iris region shape is a circular donut. Yet, the starting point for normalizing the iris region of an iris image varies due to the subject’s head tilt, distance, movement, and when the subject looks in a different direction—we call this rotational inconsistency. To overcome this rotational inconsistency between two iris templates, one template shifts two bits left or right, and the similarity score is selected from successive shifts; for the HD case, the smallest value is a successive shift value. This method may lead to an occasional matching failure because the pupil and iris may not be segmented to the exact circle boundaries—especially when the image has significant noise from motion blur or inconsistent lighting conditions. In reality, the iris outer boundary is not uniquely-defined due to its gradual gradient change from the iris to the sclera. Figure 33 illustrates a case of a less-than-optimal segmentation of the pupil and iris boundaries for an image taken in less-constrained environmental condition.

In Fig. 33, the white dotted circles outline the actual pupil and iris boundaries. The red circle is the incorrect pupil boundary segmented by the algorithm. Similarly, the green circle illustrates the inaccurate segmentation for the iris boundary.

**Fig. 34:**
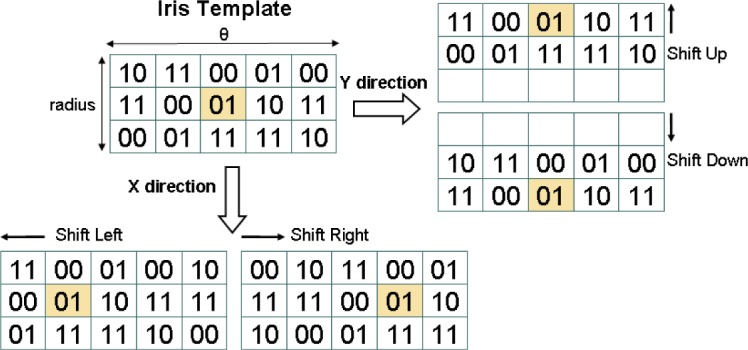
Similarity distance shifting process for X horizontal (left and right) and Y vertical (upward and downward) directions.

As a consequence, VASIR developed a new shifting method in which the template is shifted not only left and right (horizontal) bit-wise, but also upward and downward (vertical); the values for these horizontal and vertical direction shifts are indicated by X and Y, respectively. Figure 34 shows the detailed shifting process for similarity distance metrics.

The top, left iris template shows an example of the bit representation of the original iris template. When shifting the template horizontally (X), the two-bit pairs are shifted towards the left or the right. Since the horizontal bits embody angular 2π, the bits are carried over—see “01” marked with the orange shade on the left bottom side. On the other hand, when shifting vertically (Y), the bits are not carried over since they embody the radius of the circle; the top or bottom rows will be not set—see “01” marked in orange and empty pixels on the right top side.

Further, the template can be shifted in horizontal (*X*) “or” vertical (*Y*) direction and a successive shift value is selected from the values of X or Y (*XorY*). Finally, the template can also be shifted in two directions (horizontal “and” vertical) at the same time (*XandY*). For instance, when one template is shifted by one bit to the left, it also can be shifted one bit up. This new matching method can help to cope with both the rotational inconsistency and the segmentation inaccuracy.

For evaluation, we examine on the question of which similarity metric shifting method works best for VASIR performance. The complication is that VASIR is a broad-based system accommodating:

1. classical-still to classical-still (SS) matching scenario between the query set and the target set,
2. distant-video to classical-still (VS) matching scenario, and
3. distant-video to distant-video (VV) matching scenario.

The question arises as to whether a single shifting method out of the four approaches is best for all three scenarios, or whether the shifting method is scenario-specific.

**Fig. 35:**
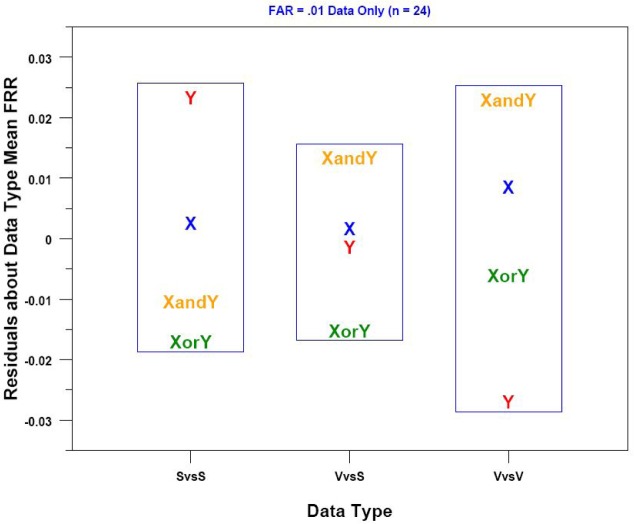
VASIR comparison of four shifting methods using the HD case for each of three scenarios (SS, VS, VV) with the residual mean FRR at FAR of 0.01 (smaller is better).

Figure 35 illustrates the HD case comparison of the X, Y, XorY, and XandY shifting methods as described above—for both the left and right eye based on the MBGCv2 distant-video dataset.

The criterion for this evaluation is the mean FRR (False Reject Rate) value at FAR of 0.01; a smaller FRR value is preferred, which means that bottom-most values yield the best performance. For the SS scenario, the results of the shifting method are ordered (best to worst) as XorY, XandY, X, and then the Y method. For the VS scenario, XorY also leads with the best results followed by the Y, X, and XandY. Interestingly, for the VV scenario, Y is better (opposite to the results of SS) and then XorY, X, XandY follow in order. Overall, XorY is robustly best because XorY is the best or second best item in all three matching scenarios.

## 6. Performance Evaluation

To provide a performance benchmark baseline, we evaluated VASIR with three datasets that were taken by multiple media source under variety of imaging conditions:

1. MBGC NIR face-visible video (distant-video) dataset,
2. All Frames subset from MBGC NIR face-visible video, and
3. Iris Challenge Evaluation (ICE) 2005 dataset.

The MBGC NIR face-visible video samples were captured with a video camera by the Sarnoff IOM system. The quality is fairly poor due to subject movements and distance. The All Frames dataset contains iris images that were automatically extracted by VASIR’s eye detection/extraction algorithm from the distant-videos. Since the data was collected while a person walks through a portal under less constrained conditions, the images are of extremely poor quality. On the other hand, the ICE2005 dataset consists of traditional still iris images, taken under constrained conditions.

### 6.1 MBGC NIR Face-Visible Video Dataset (Distant-Video)

This MBGC NIR face-visible video dataset consists of video sequences capturing a subject’s face while s/he was walking through a portal at normal walking speed from 10 feet away. The poor quality of these videos is due to motion blur, poor illumination, off-angle viewing, varying iris resolution, subject movement, subject distance, blinking, and isolating the iris region from a face/body visible video.

VASIR’s Video Processing module overcomes difficulties when dealing with such video sequences that include other features (e.g., face, hair, neck, ear) beside the eye region. As described in Sec. 4, the module consists of sub-components: Eye Region Detection/Extraction, Image Quality Assessment, and Best Quality Image Selection (See details in Sec. 4).

The MBGC NIR face-visible video dataset contains a total of 628 videos. Out of the 628 videos, 30 videos are either no eye visible or only one eye visible. Out of the 598 (= 628 − 30) videos, VASIR successfully detected the eye region in 586 videos—detailed results are provided in Table 3.

VASIR calculated the quality score for all frames using our Automated Image Quality Measurement (AIQM) algorithm, and then selected 1,172 (586 × 2 for left and right) iris images as the best quality image for the 586 videos by applying our Automated Best Image Selection (ABIS) algorithm.

**Fig. 36:**
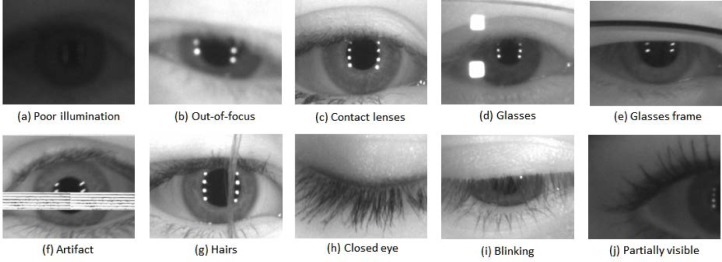
Demonstration of poor quality images selected as the best image from a video.

Although the ABIS algorithm filters out most of the extremely poor quality frames, some of best images are still of poor quality as illustrated in the examples in Fig. 36. The reason is that all frames of a video sequence had (a) poor illumination, (b) out-of-focus, or (j) partially visible, or across all frames within a video have occlusions such as (d) glasses glares, (e) glasses frames, (f) artifacts, or (g) hairs. These demonstrate the level of difficulty for iris recognition in our study.

Next, we describe a brief protocol that we used for our evaluation. The *Gallery set—*also known as the *Query set*—is the biometric sample database of known individuals for a specific implementation or evaluation experiment, and the *Probe set—*also known as the *Target set—*is the submitted biometric sample to compare against one or more references in the gallery [47].

**Fig. 37:**
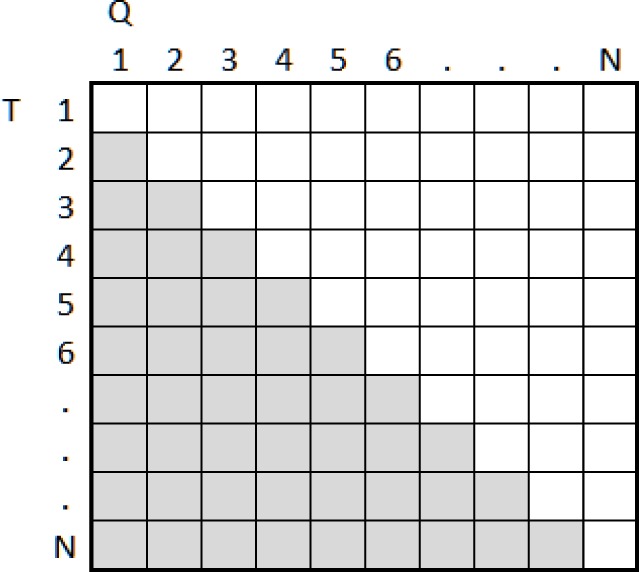
The calculated area (gray color) for the similarity matrix for matching and non-matching scores.

For benchmarking purposes, we chose to have both the query set and the target set to be the same in our study, thus we need to calculate only half of the off-diagonal elements from the similarity matrix as shown in Fig. 37 (see the shaded area)—the remaining elements being mirror-image identical.

In this study, we used the matching scores as thresholds to plot the Receiver Operating Characteristic (ROC) curves for our performance evaluations. For non-matching scores, the target images could be compared against all query images whereby the query *subject* is a different person. This full matching (one to all others) can be time-consuming and is not necessary since the non-matching scores are sufficiently consistent for virtually all query subjects. We therefore randomly selected a representative subsample of queries, all of a different person for getting the non-matching results; we found *50 query images* to be a sufficient subsample [48]—a different size subsample may be required depending on the number of subjects and images. This not only reduced execution time but also allowed us to characterize the performance more easily. Hence, for each left and right evaluation, the VASIR system compared a query image to a target image to produce the similarity scores for the 1,594 matching scores and the 29,300 non-matching scores with a randomly selected subsample of size 50 out of the 129 subjects in the 586 images.

**Fig. 38:**
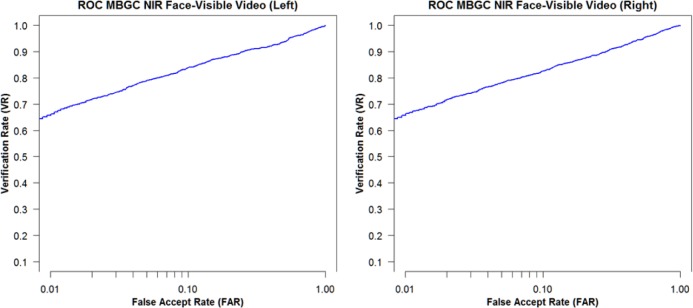
VASIR performance using MBGC NIR face-visible video (distant-video) dataset for left and right.

The ROC curves for the left and right eye using the distant-video dataset are shown in Fig. 38, and VASIR performance for iris verification is evaluated by considering two criteria:

1. Verification Rate (VR) at a fixed value (0.01) of the False Accept Rate (FAR) and
2. Equal Error Rate (EER) value where FRR and FAR met.

Conclusion-wise, when we used all components of VASIR, the VASIR performance for iris recognition had a VR of 65.8 % for left and 65.7 % for right (at FAR=0.01), and an EER value of 13.9 % for left and 14.5 % for right.

### 6.2 All Frames Dataset from MBGC NIR Face-Visible Videos

**Fig. 39:**
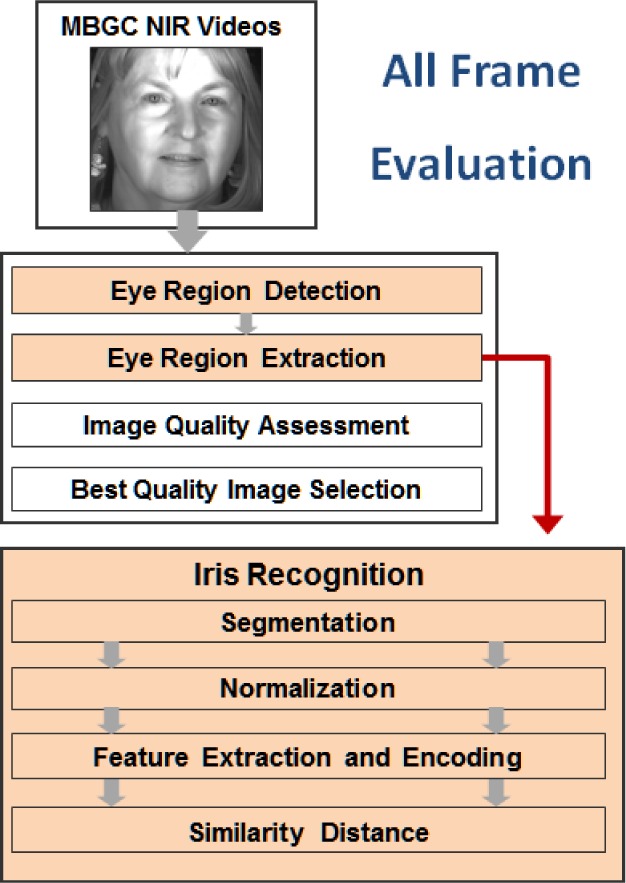
All frame evaluation for iris recognition after applying VASIR’s eye region detection and extraction.

VASIR has a component to detect the eye region from a video that contains other features such as face, hair, ear, neck, shoulder and among others. The All Frame dataset is a subset of extracted iris images after only executing the VASIR’s eye region detection and extraction components; see in Fig. 39.

**Fig. 40:**
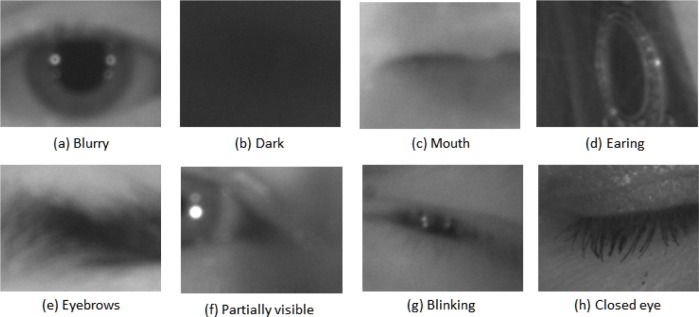
Examples of challenging image conditions for iris recognition in our study.

Out of 586 NIR face-visible videos, VASIR extracted 9,592 (4,796×2 for left and right) iris images (see details in Table 4). This subset contains extremely challenging images for iris recognition as illustrated in Fig. 40.

For example, Fig. 40 (a) shows a poor quality image due to motion blur or out-of-focus since the video was taken while a subject was moving at a distance. The proportion of blurry images within the dataset as a whole was more than 32 %. The proportion of dark images (b) in which an eye can be barely identified due to poor illumination was more than 8 %. Images like (g) or (h) with blinking or closed eye motion amount to about 2.2 % and images with only a partially visible eye region (f) were approximately 1.2 %. False positive eye region detection as shown in (c) – (e) was about 0.4 %. Some of the iris regions in the images are occluded by artifacts, glass glares, or hairs.

We examined VASIR’s iris recognition performance by including all these challenging images “as-is” because one of our goals for this study is to evaluate and promote each component of VASIR which includes not only the iris recognition sub-components (and challenges) but also the eye region detection/extraction component (and challenges). VASIR’s components will provide researchers/developers not only with the opportunity to compare their system’s components against a state-of-the-art reference, but also to augment and improve each component of the VASIR system itself.

For the All Frames subset, we used exactly the same protocol as for the distant-video dataset for iris recognition performance evaluation. The VASIR system compared a query image to a target image to produce the similarity scores for the 131,498 matching scores and the 239,800 non-matching scores with a randomly selected subsample of size 50 out of the 129 subjects in the 4,796 images.

**Fig. 41:**
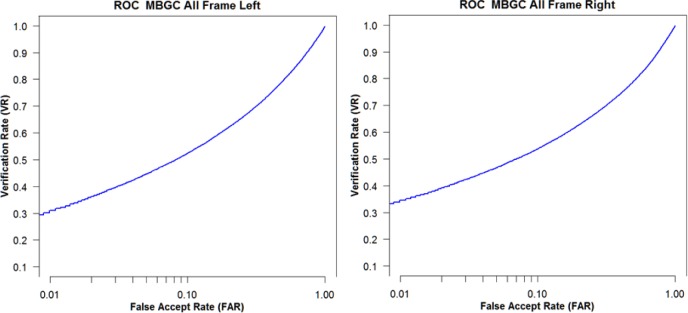
VASIR iris recognition performance using MBGC All Frames dataset for left and right.

The ROC curves are shown in Fig. 41 and the two criteria we used are as follows:

1. VR value at a fixed value (0.01) of FAR and
2. EER value.

The results show that VASIR has a VR of 31 % for left and 34.6 % for right at FAR=0.01, and VASIR’s EER performance is 30.6 % for left and 30.5 % for right.

**Fig. 42:**
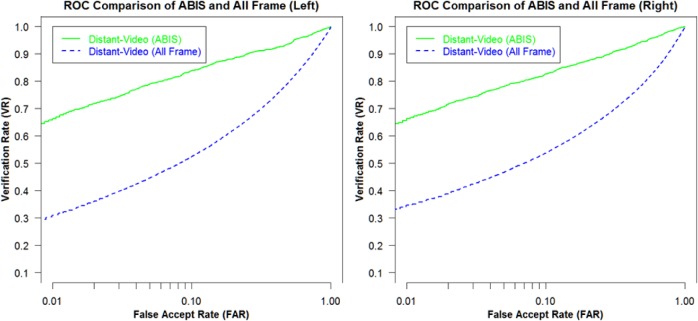
Iris recognition comparison of distant-video (ABIS) and distant-video (All Frame) for left and right.

Figure 42 shows the performance comparison between the VASIR’s complete version (namely ABIS) and all frames detected by VASIR’s eye detection/extraction component (namely All Frames).

The green solid curve indicates the results from ABIS for the distant-video dataset and the blue dotted curve indicates the results from the All Frame subset from distant-video dataset. The results indicate that VASIR’s best image selection approach (ABIS) is uniformly better (35.2 % higher performance for both left and right) than the All Frame approach. In summary, VASIR’s effort to use the best frame within a video for the matching process has proved to be not only valuable in reducing computational resources, but also to be highly effective in improving system performance.

### 6.3 Iris Challenge Evaluation (ICE) 2005

To examine VASIR’s performance using a third dataset, we measured such performance according to the ICE 2005 evaluation protocol [5]. The ICE 2005 evaluation contains Experiment 1 for the right eye consisting of 1,425 images, and Experiment 2 for the left eye consisting of 1,528 images. An algorithm compares a query image to a target image to produce the similarity scores for the 18,814 matching scores and the 1,418,317 non-matching scores.

The ICE2005 dataset contains only classic-still-based iris images since it was collected by an LG EOU 2200 system—which is a traditional iris acquisition system.

As shown in Table 5, ten entries from the academic and industrial field participated in the ICE2005 study and submitted their results with completed similarity matrices. In addition, we have included the VASIR system as the last row of Table 5. For each participant, we listed their three usage categories: 1) commercial/non-open source (blue), 2) non-commercial/non-open source (orange), and 3) non-commercial/open-source (green).

A primary motivation for the design and development of VASIR was to provide an open-source computational solution covering even the most challenging video imaging conditions. In addition, VASIR provides a solution and performs well for classic-still-based iris recognition. As shown in Table 5, out of the ten ICE 2005 participants, IrisBEE was the only *open-source* system developed for classic-still iris images.

**Fig. 43:**
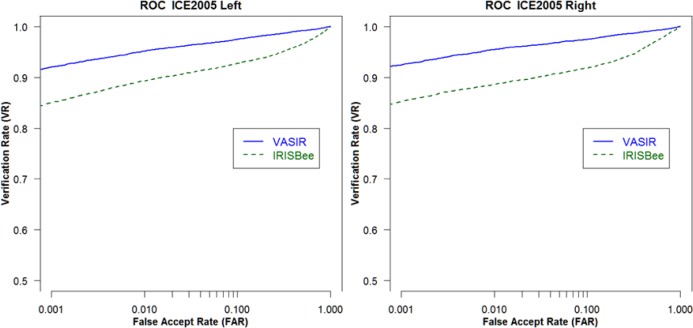
Comparison of VASIR and IrisBEE using ICE 2005 left/right (classic-still to classic-still matching scenario).

For the first evaluation, a direct comparison of the two open-source systems, VASIR and IrisBEE, was conducted using classic-still iris images (ICE2005) to gage their relative performances. Figure 43 shows a performance comparison between IrisBEE’s and VASIR’s results with the aid of an ROC curve.

The blue solid curve indicates VASIR’s result, the green dotted curve IrisBEE’s result for left/right. The results show that VASIR’s verification rate is universally better than IrisBEE over the entire range of FAR values.

In order to follow the ICE2005 protocol, two criteria were adopted:

1. VR value at a fixed value (0.001) of FAR and
2. EER value.

For the left eye, at a FAR of 0.001, VASIR has a VR of 92.0 % while IrisBEE is significantly smaller at 85.0 %. For the right eye, VASIR’s VR is 92.4 % while IrisBEE’s VR is 85.2 %. Hence, for both eyes VASIR is a significant improvement over IrisBEE. For the EER criterion, VASIR’s EER is also better (3.5 %) while the IrisBEE EER is approximately twice as large (8.1 %).

In conclusion, the results show that VASIR is superior to IrisBEE for classic-image-based iris recognition. Hence, even though VASIR is a multi-scenario system (dealing with both face-visible video and classical still iris images), it has proven to be superior to IrisBEE even in its intended single scenario (classical still-based) domain.

We now address the question as to how VASIR compares to other systems (commercial and non-commercial) for this traditional still iris image scenario. For this second evaluation, a performance comparison of ten participants’ systems (13 systems total) and VASIR was conducted (see Table 6).

Note that CAM (Cambridge University) submitted two algorithms and CAS (Chinese Academy of Sciences) submitted three algorithms. The Tohoku system only contains the outcome for the right eye. All of the systems in this table are classic-still-based, except VASIR which accommodates both classic-still and distant-video.

**Fig. 44:**
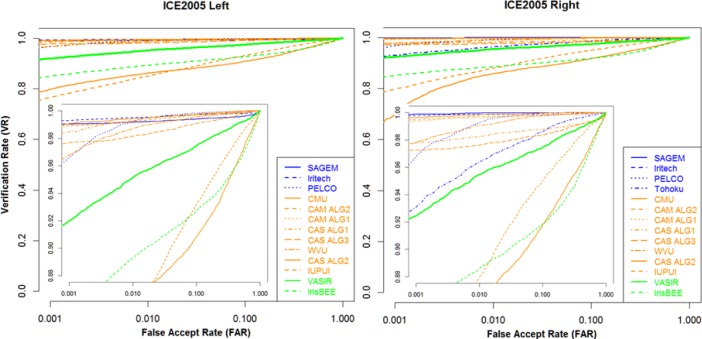
Comparison of 13 commercial/non-commercial systems and the VASIR system using ICE 2005.

We have ranked the systems within each of the three categories by VR at FAR=.001—the higher the VR of the system in each category, the better its performance. ROC curves in Fig. 44 compare the performance of the VASIR system with all of the other 13 systems for the ICE2005 left/right.

The blue curves indicate the outcomes of the commercial/non-open source systems, the orange curves of the non-commercial/non-open source, and the green curves of the non-commercial/open-source systems.

Overall, the Iritech system (blue) has the best performance out of all commercial/non-open source systems, while for non-commercial/non-open source systems the CMU system (orange) is best. For non-commercial/open-source, the VASIR system (green) has the best performance and even exceeds the performance of some non-commercial non-open source systems.

In conclusion, the results show that while VASIR was developed primarily as a tool for *distant-video-based* iris recognition, the VASIR system is a significant improvement over some non-commercial systems (e.g. CAS and IUPUI) for *classic-still-based* iris recognition as well.

This improvement is important, of course, because due to the non-commercial and open-source nature of VASIR; it may readily serve as:

1. A publically-available and best-in-class system that may be used by academia, government, and industry
2. for both benchmarking and baseline reference
3. for the promotion and advancement of multi-party algorithmic extraction, enhancement and research, and
4. not only for classic-still-based iris recognition, but also for distant-video iris recognition.

## 7. Conclusion

We developed VASIR to address the challenges and problems for iris recognition by using a structured design, analytic methodology, and rigorous orthogonal experiment design. VASIR is the iris recognition baseline that provides an open-source research platform for ideal/non-ideal image condition processing for not only still images—but also video sequences.

For the VASIR Video Processing module, we developed the eye region detection/extraction algorithm and this algorithm’s performance was evaluated using the MBGC NIR face-visible video dataset. The successful detection rate was 98 % and the failed detection rate was 2 % with the false positive rate being 0.4 %. Out of 11,341 frames in 586 videos, a total of 9,592 (4,796×2 for left and right) iris images were extracted with our pupil position alignment approach. VASIR proposed an Automated Image Quality Measurement (AIQM) and an Automated Best Image Selection (ABIS) methods and compared the ABIS approach to “ground truth” (HBIS: Human Best Image Selection). The results showed that ABIS performance was nearly equivalent to HBIS ground truth.

For the VASIR Iris Recognition module, we proposed a novel segmentation algorithm along with reduced specification of user input parameters. For the 252 left and right iris images extracted from face-visible videos, VASIR’s segmentation rate (83.7 %) was a significant improvement over IrisBEE (49.4 %). VASIR also obtained a higher performance with the optimized normalization resolution compared to a non-optimized baseline. VASIR’s noise masking scheme for feature extraction/encoding stage brought significant improvement compared to the existing “fragile bit” approach—VASIR performance increased the VR by 7 % and decreased the EER by 1.6 % for the MBGC face-visible video dataset. At the end, we examined four types of bit-wise shifting for VASIR. Our evaluation revealed that our “XorY” shifting approach—which shifts in the horizontal (X) “or” vertical (Y) direction and selects a successive shift value from the values of XorY—yielded the best results. This XorY shifting approach not only corrects the rotational inconsistency, but also segmentation inaccuracies.

To provide a benchmark baseline, we evaluated the VASIR system with three datasets taken from multiple imaging sources. For the first evaluation, we used the MBGC face-visible video dataset. At a FAR of 0.01, the VR value for VASIR performance was 65.8 % for left and 65.7 % for right and the EER value was 13.9 % for left and 14.5 % for right. For the second evaluation, we used an extremely challenging subset (namely the All Frames dataset) for iris images extracted from MBGC face-visible videos. At FAR=0.01, the VR value was 31.0 % for left and 34.6 % for right and EER was 30.6 % for left and 30.5 % for right. For the third evaluation, we used the ICE2005 dataset that contains only traditional still iris images. Following the ICE2005 protocol value of FAR=0.001, VASIR had a VR of 92.0 % for left and 92.4 % for right and the EER value was 3.6 % for left and 3.5 % for right.

Our results indicated that although VASIR was developed primarily as a tool for *distant-video-based* iris recognition, the VASIR system was a significant improvement over IrisBEE and some non-commercial systems for *classic-still-based* iris recognition as well. As such, VASIR makes for an ideal tool/resource for the iris-based biometric research community to examine the effect of algorithmic component changes, to extract and reuse the available source code, and to otherwise advance the state-of-the-art of iris recognition.

## Figures and Tables

**Table 1:** ICE2005 dataset (Classic-Still)

Position	# of Images	# of Subjects
Left Eye	1,528	120
Right Eye	1,425	124
Total	2,953	132

**Table 2:** *Distant-video* captured by an IOM system

NIR_Face_Video	Version 1	Version 2
# of total videos	149	628
# of both eyes visible	130	598
# of total frames	2,534	11,341

**Table 3:** VASIR Eye-pair detection performance for MBGCv2 distant-video

MBGCv2 distant-video	Number	Rate
Total #of eye-pair visible videos	598	100 %
Successfully detected videos	586/598	98 %
Failed detection videos	12/598	2 %

**Table 4:** A number of the extracted eye region images for MBGCv2 distant-video

MBGCv2 distant-video	Number	Rate (%)
Total # of frames out of 586 videos	11,341	100
# of false positives	21/4,796	0.4

**Table 5:** ICE2005 evaluation participants—see details in the paper by Phillips *et al.* [5]

Participants	Commercial (C) vs Non-commercial (NC)	Open vs Non-open Source	Still vs Video	Distance (Near/Far)	Acquisition Environmental Conditions
Iritech	C	Non-open	Still	Near	Constrained
PELCO	C	Non-open	Still	Near	Constrained
SAGEM(Iridian)	C	Non-open	Still	Near	Constrained
Tohoku	C	Non-open	Still	Near	Constrained
CAM	NC	Non-open	Still	Near	Constrained
CMU	NC	Non-open	Still	Near	Constrained
CAS	NC	Non-open	Still	Near	Constrained
IUPUI	NC	Non-open	Still	Near	Constrained
WVU	NC	Non-open	Still	Near	Constrained
IrisBEE	NC	Open	Still	Near	Constrained

VASIR	NC	Open	Still/Video	Near/Far	Unconstrained

**Table 6:** A performance comparison of 13 systems and VASIR using ICE2005

Systems	Com(C) / Non-com(NC)	Open / Non-open Source	Still / Video	VR|FAR=0.001		EER	

				Left	Right	Left	Right
Iritech	C	Non-open	Still	0.9917	0.9953	0.0067	0.0037
SAGEM	C	Non-open	Still	0.9904	0.9988	0.0084	0.0012
PELCO	C	Non-open	Still	0.9659	0.9679	0.0086	0.0086
Tohoku	C	Non-open	Still	NoData	0.9308	NoData	0.0263

CMU	NC	Non-open	Still	0.9907	0.9963	0.0064	0.0027
CAM ALG 2	NC	Non-open	Still	0.9893	0.9946	0.0083	0.0037
CAM ALG 1	NC	Non-open	Still	0.9888	0.9936	0.0088	0.0043
CAS1	NC	Non-open	Still	0.9848	0.9778	0.0079	0.0158
CAS3	NC	Non-open	Still	0.9767	0.9724	0.0168	0.0215
WVU	NC	Non-open	Still	0.9678	0.9784	0.0120	0.0095
CAS2	NC	Non-open	Still	0.7979	0.6991	0.0938	0.0864
IUPUI	NC	Non-open	Still	0.7668	0.7986	0.0763	0.0619

VASIR	NC	Open	Still/Video	0.9201	0.9244	0.0357	0.0348
IrisBEE	NC	Open	Still	0.8501	0.8521	0.0775	0.0841

## Footnotes

1 Certain commercial equipment, instruments, or materials are identified in this paper to foster understanding. Such identification does not imply recommendation or endorsement by the National Institute of Standards and Technology, nor does it imply that the materials or equipment identified are necessarily the best available for the purpose.

2 For detailed information about VASIR, see the website: http://www.nist.gov/itl/iad/ig/vasir.cfm.
